# A Survey on Transit Map Layout – from Design, Machine, and Human Perspectives

**DOI:** 10.1111/cgf.14030

**Published:** 2020-07-18

**Authors:** Hsiang‐Yun Wu, Benjamin Niedermann, Shigeo Takahashi, Maxwell J. Roberts, Martin Nöllenburg

**Affiliations:** ^1^ TU Wien Austria; ^2^ University of Bonn Germany; ^3^ University of Aizu Japan; ^4^ University of Essex United Kingdom

**Keywords:** CCS Concepts, • ***Human‐centered computing → Visualization techniques***, *Visualization design and evaluation methods*

## Abstract

Transit maps are designed to present information for using public transportation systems, such as urban railways. Creating a transit map is a time‐consuming process, which requires iterative information selection, layout design, and usability validation, and thus maps cannot easily be customised or updated frequently. To improve this, scientists investigate fully‐ or semi‐automatic techniques in order to produce high quality transit maps using computers and further examine their corresponding usability. Nonetheless, the quality gap between manually‐drawn maps and machine‐generated maps is still large. To elaborate the current research status, this state‐of‐the‐art report provides an overview of the transit map generation process, primarily from Design, Machine, and Human perspectives. A systematic categorisation is introduced to describe the design pipeline, and an extensive analysis of perspectives is conducted to support the proposed taxonomy. We conclude this survey with a discussion on the current research status, open challenges, and future directions.

## 1. Introduction

**Figure 1:**
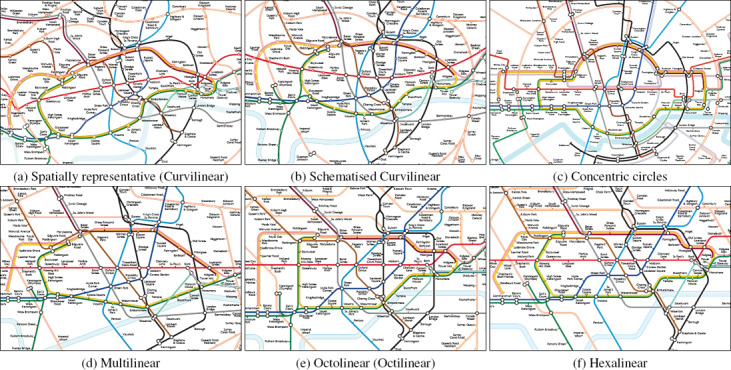
Various transit map styles of London Underground [Rob12], from less to more constrained curves or polylines in each row.

A *transit map* is a representation of a public transportation network focusing on the connectivity of stations via transit lines [Ove15], such as railway networks, bus lines, and ferry routes. Among these examples, *metro maps, subway maps*, or *tube maps* [Gar94, Ove03, Rob12] are typical transit maps of metropolitan railways. To increase legibility, designers often simplify complex structures using an abstracted representation, for example, by straightening lines and distributing stations evenly. This process of transforming a topographical map into a network diagram is called schematisation. The main components of a transit map include symbols for stations and interchanges, names, and coloured lines, linking the stations and indicating the types and ranges of transportation services. The resulting simplified, and often visually pleasing *schematic* maps capture essential structures of the transport networks, as well as the images of cities [Lyn60]. The main purpose of schematic maps is to facilitate passengers’ orientation and navigation of the transit network. Examples of the London Underground (Figure 1) demonstrate several layout styles (e.g., curvilinear, concentric circles, multilinear, etc.) applied to the same transit network. Some schematisations are optimised for usability, whereas others are created for visual entertainment (Figure 1(c)).

According to our interviews with map illustrators [WTH*13] and knowledge gained from transit map history [Gar94], a schematised map is often created in an iterative process. This goes through a bi‐directional loop between organising the information to present, designing perceptually effective visual languages, and implementing the final product. The result depends on the designer's intuition and skills in order to produce an easily understandable and visually informative map which is effective for its intended tasks. This is a time‐consuming procedure. Thus, scientists have been researching more efficient solutions in order to improve on this. Over the last two decades, transit map problems have been investigated by designers, computer scientists, psychologists, and even the general public, each with different interests and expertise.

As examples of studies, designers [Cer16, Ove08] have investigated visual languages that can be used in standard map production leading to better design strategies. Computer scientists have developed algorithms to mimic the map production procedure [WNTN19] by translating data, design criteria, and objectives into a machine‐processable form. Psychologists have researched the effectiveness of transit maps and the usability benefits of different styles (see Figure 1). In most cases, the three communities are active individually and collaborate seldom to achieve more advanced goals. In this survey, we aim to cover a full visualisation pipeline for transit maps by investigating the existing work from the (D)esign, (M)achine, and (H)uman perspectives and pointing out the existing gaps occurring in current real‐world applications.

Five earlier, but more narrow survey papers on schematic maps exist. Wolff [Wol07] summarised early techniques from an algorithmic perspective. Two surveys [Nöl14, WNTN19] investigated more recent algorithms, as well as aspects of interactivity and applications. Two more surveys [Rob14a, Gri19] summarised user performance and usability. Authors from three of the papers are coauthors of this survey. We aim to discuss a much broader model that covers the design, machine, and human perspectives of transit map layout.

We will first discuss key elements in each perspective. The goal is to investigate the current state of developed techniques, providing an overview of the scientific field, and also to identify gaps between perspectives. The discussion of fusions between them will highlight opportunities for future collaborations and unifications, facilitating discussion across multiple disciplines.

**Contributions.** The contributions and structure of this survey are:

- A unified taxonomy and overview of published work in the field of transit map layout (Section 2).
- A full summary of design principles, research focus, current achievements, and gaps in existing work from the (**D**)esign, (**M**)achine, and (**H**)uman perspectives (Sections 3 to 5).
- A discussion of potential for fusions between the three perspectives (Section 6).
- An outline of potential challenges and key research directions derived from the previous discussions (Section 7).

**Scope of the Work.** We performed an extensive search of online literature databases, covering the domains of visualisation, design studies, algorithms, and psychology to exhaustively collect relevant work to be included in the survey. Some results were removed if they did not explicitly fit within the scope. The primary focus of this paper will be on layout techniques, as this is the most researched topic, as summarised in Table 1. Within this topic, readers will find that the focus of past researchers has been on rail‐based transit. Schematisation techniques can, of course, be applied to road‐based transit networks, too, but the potential conflict between schematised lines and visible topography reduces the benefits that can be achieved. The materials of this survey have been put on the web‐page http://survey.schematicmapping.org/.

**Table 1:** Table representing the literature search and sources.

Search Domain/Method	Sources
Visualization	IEEE TVCG, CGF, IEEE Vis, Euro Vis, PacificVis, GD, etc.
Cartography & Geoinformatics	The Cartographic Journal, Geographic Information Science, Schematic Mapping Workshop, etc.
Psychology	Psychological Research, Cognitive Research, etc.
Digital Libraries	IEEE Xplore, Wiley DL, ACM DL
Keyword & Citation	Google Scholar, Book Publisher

## 2. Survey Model

**Figure 2:**
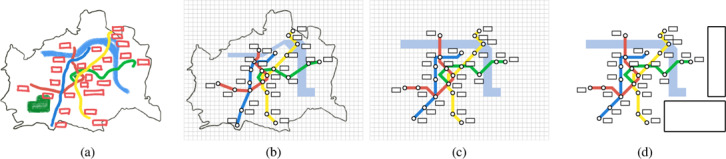
A process for transit map creation: (a) a sketch of the essential elements; (b) iterative refinement of stations and lines on grids; (c) a schematic form fulfilling aesthetic criteria; and (d) a finalised map with legends.

Manually creating a transit map is an iterative process. After an initial discussion to collate requirements with the people who commission the representation, the designer will pass through various stages [Rob12, WTH*13], including (a) a sketch of the essential elements, (b) iterative refinement of positioning of stations and lines, (c) a schematic form fulfilling aesthetic criteria, and (d) a finalised map with legends. In Step (a), a rough composition facilitates the placement of essential elements (Figure 2(a)). Besides the transit network, these might be geographical features, such as rivers and landmarks in a city. In Step (b), the designer performs an iterative adjustment to align stations and lines on grids and distribute them evenly, while preserving their relative position in comparison to other elements. He or she refines this until all criteria are fulfilled (Figure 2(b)). Once a clean layout is achieved (Figure 2(c)), the designer is ready to finalise the drawing in Step (d), by incorporating additional information, such as titles, legends, text, and images.

**Figure 3:**
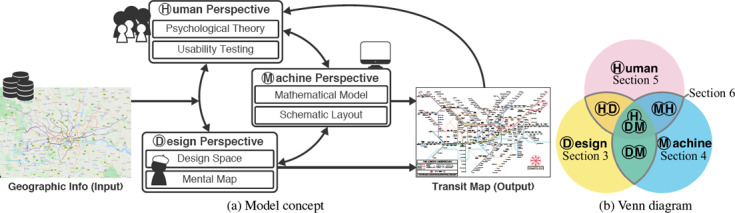
Two diagrams that describe the structure of this survey, (a) Shows how theoretical knowledge might permeate through the system, influencing the design of maps. (see Section 2). (b) Shows a conceptual diagram that depicts the coverage in the upcoming sections.

The aforementioned steps are summarised as a conceptual diagram that relates the three perspectives of this survey (Figure 3). This is built upon an integration of the map creation procedure [Cer16] and the visualisation pipeline [KMS99]. We consider a topographical map with attributes (e.g., names, landmarks, photos) as our *input*. The process flows via iterative steps (Figure 3(a)) which may involve any or all of the three perspectives. For the design perspective, aesthetic criteria are investigated. In the machine perspective corresponding algorithms are developed. In the human perspective the effectiveness of the selected criteria is validated. This continues until either an illustrator or a machine creates the output: the schematic map. For example, a map can be created by first selecting visual variables based on designers’ experience. The encoding can be converted into machine‐readable form to automatically generate the layout (Input → (**D**)esign → (**M**)achine → Output). Alternatively, an analysis of potential user tasks can be inserted (Input → (**D**)esign → (**H**)uman → (**D**)esign → (**M**)achine → Output).

Ideally, the three perspectives should not be investigated or practised in isolation since they are strongly mutually supporting. However, not every researcher or designer has the capacity to cover all perspectives, and individual perspectives are not always sensitive to input from others. One goal in this survey is also to understand the reasons why the quality of machine‐generated maps is generally held to be poorer than the human‐created maps and hence identify the tasks that are straightforward for trained humans to perform but cannot be easily formulated for a machine. The potential for interactions between perspectives is highlighted by the Venn diagram in Figure 3(b). We will present each individually, and then discuss their potential input into the other perspectives. We identified papers relevant to each perspective by researching scientific publications and books in the corresponding fields as summarised in Table 1.

Logical search operators were used in digital libraries to find directly related literature, while keyword and citation searches were performed to find other relevant works. A screening process was conducted to finalise the papers that are strongly connected to transit map layout. We removed duplicated works, for example, preliminary conference publications. We classified how relevant a paper is to each perspective, and examined how they are related to each other both by high‐level conceptualisations and detailed‐level implementations. The classification will be explained in the following sections.

## 3. Design Perspective

We surveyed 44 references to collate and categorise the most frequently‐expressed criteria for designing transit maps (see Table 2), either explicitly expressed by map designers, or inferred from their expression via visual languages. The first factor of our taxonomy refers to the design principles for layout and geometry, which are considered as *macro* (or *global)* layout parameters (Section 3.1). The second factor involves placement of text and image labels (Section 3.2), which are primarily *micro* (or *local)* design issues and are often accommodated by adjusting local geometric configurations of lines and stations. The third factor comprises frequent visual variables, such as colour assignment (Section 3.3) and the fourth concerns additional requirements for possible application scenarios such as static, interactive, and dynamic maps (Section 3.4). We conclude in Section 3.5 by providing links to the machine perspective (Section 4).

**Table 2:** Key factors of the references assigned to design perspective, which are collected from published books, survey, design study, user study, and eye‐tracking study papers. The solid horizontal lines separate the factors introduced in each subsection of Section 3, while the dashed lines separate macro and micro properties.

	Published books								Survey		Design study																								User study					Eye‐tracking study					Σ 44
	[Cer16]	[Gar94]	[Ove03]	[Ove08]	[Ove15]	[Rob19b]	[Rob05]	[Rob12]	[Ave14]	[Rob19a]	[AH06]	[ALB11]	[Bai10]	[BAQ12]	[BWNW16]	[Deg13]	[Che14]	[Car15]	[CL19]	[CSS19]	[DK09]	[DK10]	[FC14]	[FWD*17]	[Gol65]	[Llo17]	[XY09]	[LRR18]	[Mer13]	[Rob10]	[Rob14a]	[Rob14b]	[Sch14]	[MV83]	[GPB16]	[GW11]	[GZW*17]	[SWKE17]	[Ver08]	[Bur16]	[Bur18]	[BKM18]	[BRB*14]	[NOK*17]	
**D1 Layout and geometry**																																													
D1.1 Layout types																																													
D1.1.1 Geographic			×	×	×	×	×	×												×																	×								8
D1.1.2 Curvilinear	×		×		×	×	×	×					×																	×	×	×													10
D1.1.3 Concentric circles			×		×	×	×	×																								×						×							7
D1.1.4 Multilinear			×	×	×	×	×	×																						×	×	×													9
D1.1.5 Octolinear	×	×	×	×	×	×	×	×			×	×	×	×	×	×		×	×				×			×		×	×	×	×	×	×	×			×		×	×	×	×		×	31
D1.1.6 Tetralinear			×		×	×	×	×																																					5
D1.1.7 Others			×	×	×	×	×	×									×																			×									8
D1.2 Relative position of stations	×	×	×	×	×	×	×	×		×		×	×	×	×	×	×	×	×		×	×	×	×	×	×		×	×	×	×	×	×	×	×	×	×	×	×	×	×	×	×	×	40
D1.3 Global scale distortion	×	×	×	×	×	×	×	×		×	×	×	×	×	×	×	×	×	×				×	×		×		×	×	×	×	×	×	×	×	×	×	×	×	×	×	×	×	×	38
D1.4 Spatially‐separated stations	×	×	×	×	×	×	×	×			×	×			×	×	×	×	×				×			×		×	×	×		×	×	×			×	×	×	×	×	×		×	30
D1.5 Even spacing of stations	×	×	×	×	×	×	×	×		×	×	×		×	×	×		×	×				×			×		×	×	×		×	×	×			×		×	×	×	×		×	30
D1.6 Simplification of trajectories	×	×	×	×	×	×	×	×	×	×	×	×		×	×	×	×	×	×				×			×		×	×	×	×	×	×	×			×	×	×	×	×	×		×	34
D1.7 Symbolic shapes	×								×	×	×			×				×											×			×	×					×	×						11
D1.8 Co‐routed lines	×	×	×	×	×	×	×	×		×	×	×		×	×		×		×							×		×	×	×		×	×	×			×		×	×	×	×		×	28
D1.9 Zone partitioning			×	×	×	×	×	×									×													×							×		×					×	11
**D2 Text and image labels**																																													
D2.1 Consistency	×	×	×		×	×	×	×		×	×	×			×	×		×					×			×		×	×	×		×	×	×					×	×	×	×		×	26
D2.2 Label proximity				×																			×																						2
D2.3 Overlap‐free labels	×	×	×	×	×	×	×	×				×			×	×	×	×					×			×		×	×	×		×	×	×					×	×	×	×		×	26
D2.4 Name orientation																																													
D2.4.1 Horizontal name		×	×	×	×	×	×	×		×		×			×	×	×			×			×			×		×	×	×		×	×	×					×	×	×	×		×	26
D2.4.2 Diagonal name			×		×					×													×							×															5
D2.4.3 Vertical name			×		×		×			×													×																					×	6
D2.4.4 Perpendicular to line			×		×					×													×																						4
D2.5 Typography			×	×	×	×	×	×		×		×	×		×	×	×	×		×			×			×		×	×	×	×	×	×	×		×	×		×					×	27
**D3 Visual variables**																																													
D3.1 Colour																																													
D3.1.1 Predefined colour	×	×	×	×	×	×	×	×		×	×	×			×	×	×	×	×	×					×	×	×	×	×	×		×						×	×	×	×	×		×	30
D3.1.2 Optimized colour																								×																					1
D3.2 Line styles	×		×	×	×	×	×	×		×	×	×			×				×															×					×					×	15
D3.3 Station styles	×	×	×	×	×	×	×	×		×	×	×			×	×	×			×			×			×			×	×		×	×	×			×		×	×	×	×		×	28
D3.4 Landmark									×																																				1
**D4 Application scenario**																																													
D4.1 Static	×	×	×	×	×	×	×	×		×	×	×	×	×	×	×	×	×					×		×	×	×	×	×	×		×	×	×			×	×	×	×	×	×	×	×	35
D4.2 Interactive									×				×						×	×																									4

### 3.1. Layout and Geometry

Design criteria for schematic maps have been discussed in several references [ALB11, BAQ12, Deg13, BWNW16, Rob19a] and published books [Cer16, Gar94, Ove03, Ove08, Ove15, Rob05, Rob12, Rob19b]. We identified the most frequently mentioned criteria in the references and determined whether they were relevant to global or local properties.

**Figure 4:**
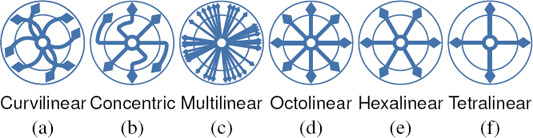
Layout types of a transit map.

**Layout Types.** At an early stage of evolution of transit maps, *geographical* layouts were iteratively simplified by smoothing off minor topographical details from the lines, commencing the pathway towards abstracted representations. These *curvilinear* (Figures 1(a), 1(b), and 4(a)) layouts facilitated making sense of complex transit networks. For example, many such maps were created for the London Underground network from the 1920s to the early 1930s [Gar94, Rob12]. More recently, *concentric circles* (Figures 1(c) and 4(b)) can be thought of as a sophisticated version of the curvilinear style, in which lines and stations are constrained as an ortho‐radial layout of orbits and spokes. Curvilinear styles were superseded by polyline‐based layouts to further simplify configurations. With these, irregularly‐shaped geographical lines are straightened to enhance legibility. A *multilinear* map (Figure 1(d) and 4(c)) is the most relaxed version in the sense that it allows any angle of lines to be used [RGL17]. More constrained is the *k‐*linearity setting, where angles between adjacent edges associated to a station are equal to 360°/2*k* [NN19]. This means that the corresponding *k*‐linearity design becomes more restricted as *k* reduces. For example, *k* is equal to 4, 3, and 2, in Figures 4(d) *(octolinear)*, 4(e) *(hexalinear)*, and 4(f) *(tetralinear)*, respectively. The intention is to reduce the number of unwanted changes in the direction of transit lines so that the visual tracking of users is not disrupted.

Among the possible *k*‐linearity designs, the traditional *octolinear* layout (see Figure 1(e)) has become a de‐facto standard, first being adopted in 1931 for the Berlin S‐Bahn network, then by Henry Beck for the first London Underground diagram in 1933. For octolinear designs, only four equally spaced angles are permitted for the alignment of lines, i.e., horizontal (0°), vertical (90°), and two diagonals (45° and 135°). *(In this paper, we adopt the term octolinear instead of octilinear* [NW11] *to refer to the* 4‐*linear design, thus retaining the consistency of the Greek prefixes.)*

Each layout type has its own merits. Roberts [Rob10] made a preliminary report on possible layout designs of the Washington, DC metro network, in which he composed schematic maps of different linearity together with a curvilinear map and discussed their pros and cons. Roberts [Rob14b] also performed an Internet study on the design preferences of the general public by preparing nine possible layouts of transit networks with three design rules (curvilinear/multilinear/octolinear) and three design priorities (geographical/simple line trajectories/complex trajectories), and discovered that the linear designs were rated as being more usable then curvilinear ones, with octolinear ones being preferred by far, and also that simple line trajectories were important to users. Examples of different layout types are shown in Figure 1.

**Relative Position of Stations.** Many users complain if the relative positions of pairs of stations are considerably distorted, for example reversing their north‐south relationship, and Roberts et al. [Rob14a, RGL17] have discussed the importance of topographical accuracy in schematic representations, for example, in terms of conflicts with user mental models of a city. Designers therefore need to be aware that relative station positions should be preserved unless other benefits of schematisation outweigh this.

**Global Scale Distortion.** Map designers often enlarge the central downtown area compared to suburban areas because, for example, dense regions of the network can cause difficulties in labelling stations [Ove08, Sch14]. This reduces visual clutter arising from such congestion and improves legibility of the most important region of the map [BRB*14].

**Spatially‐separated Stations.** Different stations must be placed in different locations. This rule is inherent to a schematic representation to prevent any station from occluding another.

**Figure 5:**
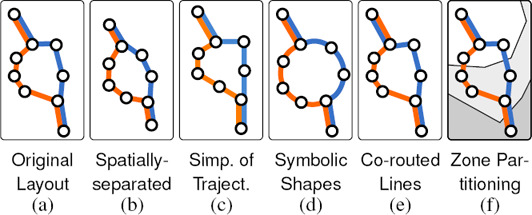
Factors influencing original geographical structures.

**Even Spacing of Stations.** It is often recommended that distances between adjacent stations are equalised along lines. This criterion can facilitate a compact representation and further lead to an organised *grid* alignment. It can also enlarge a complex dense central area with respect to suburbs. Degani [Deg13] and Lloyd [Llo17] explored a close connection between aesthetic design inherent in the grid structure of the Beck‐style layout and modern art, including paintings drawn by Piet Mondrian. Figure 5(b) illustrates equalised distances between the stations of Figure 5(a).

**Simplification of Trajectories.** It is important to aim for simple line trajectories across the map, i.e., reducing the number of changes in direction. This will minimise visual disruption when tracking lines. However, preservation of topographical accuracy, as pointed out by Roberts [Rob14a], is a conflicting factor in the design of schematic maps. This leads to the need for exploring the best compromise between topographical/geographical and schematic/simplified representations (see Figure 5(c)).

**Symbolic Shapes.** Highlighting transit lines that have specific topology, such as circular routes, can also increase the visual legibility of the entire network. For this purpose, we often schematise such routes as *symbolic shapes;* for example, transforming an irregular circular path into a geometric circle (Figure 5(d)).

**Co‐routed Lines.** There are several choices for rendering multiple lines if they share consecutive stations. For example, multiple lines can be tightly bundled without gaps between them, or we can space adjacent lines to emphasise their separateness. This has an effect on the perception of line crossings, see Section 4.2.4. The layering of co‐routed lines can also be changed according to their connectivity to the stations in their neighbourhood as shown in Figure 5(e).

**Zone Partitioning.** In many cities, we need to overlay the transit network over *fare zones*, e.g., in London Underground maps. In this case, we can simplify the depiction of the transit network by exploring the best compromise between the visual clarity of the network topology versus the underlying zones. Because fare zones often have a geographical basis, their inclusion may entail a reduction in topographical distortion of the schematic.

### 3.2. Text and Image Labels

Proper placement of text and images is a key factor in ensuring that a map is legible. Here, we enumerate the major criteria for ensuring the visual quality of schematic representations.

**Figure 6:**
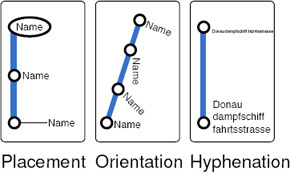
Labelling styles.

**Consistency.** Annotating a station node with its name is an essential requirement (see Figure 6). For *internal labelling*, station names are placed in the vicinity of the node, or inside it. In contrast, for *external labelling*, names are placed around the boundary of the map and often connect to the node with a leader line. Generally, it is necessary to select one of the two sides of each transit line when we embed station names. *Consistent* placement is considered preferable, in which all station names are on the same side of a line. Otherwise, placement of station names is *inconsistent*.

**Label Proximity.** When placing station names at a node, especially in the context of internal labelling, it is preferable to minimise the distance between the station node and its name to enhance the *perception of proximity*.

**Overlap‐free Labels.** Non‐overlapping station name labels are essential for legibility [Yoe72, Imh75]. This requires sufficient labelling space for station names, especially in a downtown area, where transit lines are dense and multiple station labels are more likely to intersect.

**Name Orientation.** For better readability, station names are aligned *horizontally* as often as possible. However, orientation can be switched to diagonal or vertical if horizontal placement incurs conflicts with other station names (see Figure 6). A useful heuristic is to align the station names such that they are perpendicular to the corresponding line [Ove03, Ove15, Rob19a].

**Typography.** Typographic principles have a substantial impact on legibility [MV83, Rob 14b, Rob 19a]. This often requires a compromise between the choice of letters for station names and the layout of transit lines, especially for long station names in congested downtown areas. Condensed typefaces can alleviate this problem but another solution is to break a station name into multiple lines, applying standard *hyphenation* techniques to make them as compact as possible without violating linguistic form (see Figure 6).

### 3.3. Visual Variables

In this section, we cover other important visual variables for schematic transit maps, such as colour assignment, style selection of transit lines and station nodes, embedding of landmark symbols, etc.

**Colour.** Colour is one of the most prominent visual variables and effectively discriminates between transit lines. Several eye‐tracking studies [NOK*17, Bur18] have demonstrated that coloured maps facilitate visual interpretation. Goldstein [Gol65] devised a colour coding scheme for composing transit maps and Liu and Lin [XY09] highlighted the importance of colour harmony principles. Lloyd et al. [LRR18] categorised colour assignment schemes into several types, as detailed in Section 5.2.4. It is beneficial to maximise the *perceptual distance* between every pair of transit line colours through colour‐map optimisation [FWD*17]. Online tools [HB03] may be employed for finding the best choice among pre‐defined colour maps.

**Figure 7:**
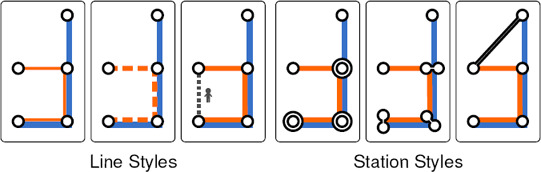
Possible line and station styles.

**Line Styles.** Line styles can represent *service plans* along fixed routes, such as express versus local trains, regularity, frequency, capacity, etc. (see Figure 7). Typical examples of styles include adjusting the width of the lines, choosing between solid, hollow, and dashed patterns, and so forth. Transit lines under construction can be represented by broken line styles or tints of their assigned colours. Additional line style examples can be found in [AH06].

**Station Styles.** Node styles for stations can be varied by size, shape, and silhouette width, for example, adjusted according to the type and importance of the corresponding station (see Figure 7). More salient node styles can indicate *interchange stations* and *terminals*, discriminating such stations from ordinary ones. Specific station styles are also listed in [AH06].

**Figure 8:**
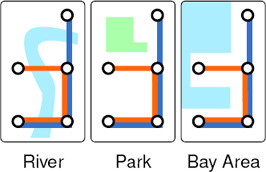
Landmark examples.

**Landmarks.** Embedding representative landmarks may augment the understanding of the context of the map. Examples include *topographical features* such as mountains, rivers, bays, etc. and *areas/sites of interests* including gardens, monuments, museums, stadiums, towers, etc. The shapes of the topographical features should be simplified to match the visual language of the schematic, and positioned such that correct nearby stations can be identified, as discussed in [Ave14]. Figure 8 presents examples.

### 3.4. Application Scenario

The consideration of design criteria also depends on the possible applications in terms of visualisation models such as static, interactive, and dynamic maps.

In general, a schematic transit map is created as a *static* representation, in which we mostly employ a top‐down design approach in the sense that we first introduce macro layout rules and then impose micro layout constraints on the map. Despite the limitation of scale and range imposed on static maps, Bain [Bai10] enumerated typical design rules to enhance the visual quality of printed maps as opposed to interactive versions.

People can also use *interactive* tools for assembling travel routes, which can create customised schematic maps. In the latter case, these are more likely to use a bottom‐up approach by incorporating micro layout methods first. Cousins et al. [CSS19] developed a framework for interactively designing traveller‐centric transport maps by rearranging layouts according to selected transport modes. Craig and Liu [CL19] employed a combination of mobile devices and large display interfaces to help travellers interactively design their journey plans on transit maps.

Recent advances in digital technology enable applications that adjust transit maps dynamically as users explore them interactively. In this case, layouts may dynamically change according to the navigation context associated with the current position and scale [WTPA17b, WTPA17a]. In such circumstances it is desirable to maintain a topographically consistent placement of components.

### 3.5. Links to the Machine Perspective

The common use of the design rules developed by Beck has led to the conjecture that octolinearity is *a schematic mapping gold standard* [Mer13, RNL*13]. In practice, Beck's design principles have been widely accepted by the public, and the readability of the associated map representations has been confirmed in several eye‐tracking experiments [BRB*14, Bur16, BKM18]. Nonetheless, in a qualitative survey of experts in geospatial science [Car15], Beck‐style maps were considered to be not necessarily the best for all purposes. A study of existing schematic maps [MV83] revealed eight parameters that characterise the legibility of Beck's maps and also identified unwanted cases in which Beck's method fails to improve the layout of transit networks in major cities. Schwetman [Sch14] listed the pros and cons of Beck's designs through an analysis of post‐Beck transit maps. Field and Cartwright [FC14] discussed possible risks hidden behind the overuse of Beck styles, which may lead to an *out‐of‐date* design that limits map usability.

This suggests the need for exploring new design principles that are potentially superior to those of Beck‐style schematic maps [Che14]. In this sense, layout algorithms, which will be detailed in the next section, can offer new forms of schematic map design and customisation, even though they may not produce the iconic representations manually composed by map designers. Thus, the design and machine perspectives can mutually support each other by providing ideas and tools that enhance the quality of such schematic maps.

In the design perspective, we described criteria according to illustrators’ preferences, which are conceptual and often laborious to implement. To reduce these demands, the criteria can be transformed into a formal language that can be processed by a machine. However, this is ongoing: while many criteria have been translated for the machine perspective, others have not yet been taken into account.

## 4. Machine Perspective

**Figure 9:**
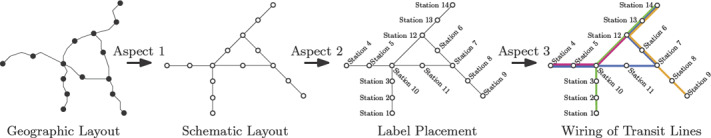
From the machine perspective the creation of a transit map consists of three algorithmic aspects.

In this section we first present a formal framework that unifies preceding models and then we illuminate the development of algorithms. The framework, which we describe in Section 4.1, is based on several existing models [NW11, Nol14, NH18, WNTN19] and is intended to summarise and unify the taxonomy. Still, as a generic framework, it does not formalise all details, but allows the reader to fill the gaps for their own purposes. In particular, we have chosen a technical and mathematical description so that readers can use this model as starting point for developing their own algorithms. We discuss 46 references that consider the creation of transit maps from the machine perspective (see Table 3). We distinguish three major algorithmic aspects when creating a transit map (see Figure 9). The first one deals with creating the layout of the network. The second aspect then takes the placement of station labels into account. Finally, the third aspect considers the routing and ordering of transit lines along the edges of the network. Afterwards, in Section 4.2 we discuss the state of the art with respect to the above three algorithmic aspects. In Section 4.3 we present results based on user studies and benchmarks, respectively.

**Table 3:** Key properties of the references assigned to the machine perspective. The references are partitioned into groups that consider the labelling and layout problem in an integrated way, successively as well as solely. The last group includes the references considering the wiring problem.

	Layout																				Labelling				Layout and Labelling (Integrated)							Layout and Labelling (Successive)							Wiring								Σ 46
	[Ave07]	[AAWJ07]	[AM00]	[BLR00]	[CdBvK05]	[CR14]	[vDL18]	[vDvGH*14]	[DHM08]	[FHN*12]	[LD10]	[MG07]	[MSBH12]	[Ney99]	[NN19]	[OS15]	[TL14]	[TLX15]	[WR13]	[WTAT06]	[GIM*01]	[NH18]	[WTLY11]	[YMK*18]	[CR13]	[CR11]	[CR15]	[NW11]	[OMI18]	[SR05]	[SRMOW11]	[HMdN06]	[TMK*19]	[WC11]	[WP16]	[WPT*15]	[WTH*13]	[WTLY12]	[ABKS10]	[AGM08]	[BBS19]	[BNUW06]	[FP13]	[FPW15]	[Nöl10]	[PNBH16]	
**M1 Layout properties**																																															
M1.1 Layout type																																															
M1.1.1 Any type																					X	X	X																X	X	X	X	X	X	X	X	11
M1.1.2 Multilinear									X			X		X	X				X		X	X	X														X		X	X	X	X	X	X	X	X	17
M1.1.3 0ctolinear	X	X	x		X	X	X		X		X	X	X	X	X	X	X	x	x	X	X	X	X	X	X	x	X	X	X	X	X	X	X	x	X	X	X	X	X	X	X	X	X	X	X	X	43
M1.1.4 Curved								X		X											X	X	X												X				X	X	X	x	x	X	X	X	14
M1.1.5 Others				X																	X	X	X																X	X	X	x	x	X	X	X	12
M1.2 Topology (L1)	X	X	X	X	X	X	X	X		X	X		X		X	X	X	X	X			X	X	X	X		X	X	X			x	X	X	X	X	X	X									30
M1.3 Straightness (L2)						X		X	X	X			X		X	X									X	X	X	X	X	X	X		X	X	X	X	X	X									20
M1.4 Distortion (L3)		X			X			X	X		X	X		X			X	X	X	X									X				X	X	X	X		X									17
M1.5 Edge length (L4)						X	X	X		X			X		X	X			X			X			X	X	X	X	X	X	X	x		X	X	X	X	X									22
**M2 Labelling properties**																																															
M2.1 Disjoint labels (P1)																					X	X	X	X			X	X	X				X			X	X	X									11
M2.2 Adjacent labels (P2)																					X	X		X	X	X	X	X	X	X	X	x	X	X	X		X										15
M2.3 Remote labels (P3)																							X													X	X	X									4
M2.4 Consistency (P4)																						X			X	X	X	X	X	X	X	X		X	X												11
M2.5 Name orientation (P5)																																															
M2.5.1 Horizontal																					X	X	X	X	X	x	X	X	X	X	X	X	X	X	X	X	X	X									18
M2.5.2 Vertical																						X		X								x	X		X												5
M2.5.3 Diagonal																						X		X	x		X	X	X			x	X	X	X												10
M2.5.4 Others																						X										X															2
M2.6 Layout compliant (P6)																						X	X	X				X	X				X			X	X	X									9
**M3 Wiring properties**																																															
M3.1 Station crossing																																									X						1
M3.2 Connection crossing																																							X	X		X	X	X	X	X	7
**M4 Algorithms**																																															
M4.1 Formal objective		X			X		X		X			X	X	X	X	X			X		X	X	X	X	X	X		X	X	X	X		X	X	X	X	X		X	X	X	X	X	X	X	X	33
M4.2 Exact		X			X							X	X	X	X	X					X	X						X					X			X	X	X	X	X	X	X	X	X	X	X	22
M4.3 Heuristic	X	X	X	X		X	X	X	X	X	X	X	X				X	X	X	X		X	X	X	X	X	X	X	X	X	X			X	X			X		X	X			X			32
**M5 Evaluation**																																															
M5.1 Implementation	X	X	X	X	X	X	X	X	X	X	X	X	X		X	X	X	X	X	X		X	X	X	X	X	X	X	X	X	X	X	X	X	X	X	X	X			X					X	38
M5.2 User study/Expert interview						X					X						X	X										X		X	X		X		X		X	X									11

### 4.1. Mathematical Framework

We assume that the structure of the transit network is given as a (geometrically embedded) graph *N* = *(S,C)* with vertex set *S* and edge set *C* such that each vertex corresponds to a station in the network and each edge represents a direct connection between two stations. Further, we are given a *path cover T* of *N*, i.e., a set of paths in *N* representing the different transit lines, such that each connection *e* € *C* belongs to at least one path in *T*. Based on an initial geographical layout of *N* and *T* we aim at creating a schematic transit map *𝕄*. Formally, we interpret *𝕄* as a collection of geometric primitives such as points, lines and simple polygons in the plane representing *N* (and including additional information such as labels and transit lines). For the following description we define that a *section* of *N* is a maximally long path in *N* that only consists of degree‐1 and degree‐2 vertices.

#### 4.1.1. Algorithmic Aspect 1 – Layout

A *layout 𝕃* of *N* is a mapping that assigns to each vertex *s* € *S* a point *p* € ℝ^2^ and to each edge *e* € *C* a curve connecting its incident vertices. We say that *p* is a *station in 𝕃* and *c* is a *connection in 𝕃*. Mathematically, we distinguish three *styles* of layouts.

1. *Multilinear layout*. Each connection is represented as a polyline whose segments are parallel to some orientation from a specified set C of orientations (also known as *C‐oriented* layout [Ney99]). Important special cases are octolinear, hexalinear and tetralinear layouts, recall Figures 1(d)–(f) and 4(c)–(f).
2. *Curvilinear layout*. Each connection is represented by a smooth curve, e.g., a Bézier curve or a circular arc (see Figures 1(a)–(b) and 4(a)).
3. *Concentric layout*. Each connection runs along an ortho‐radial grid that consists of a set of concentric circles and rays emanating from the centre of the circles (see Figures 1(c) and 4(b)).

We denote multilinear, curvilinear, and ortho‐radial layouts as *schematic layouts* of *N* (see Figure 1 for examples). In graph drawing, concentric layouts also became known as *ortho‐radial graph drawings* [NRW19]. Moreover, a layout is a *geographical layout* if each vertex of *S* is placed at the properly projected position of the geographical location of its station and each edge {*u, v*} € *C* is represented by a curve (typically a polyline) that describes the geographically correct course of the connection between the stations of *u* and *v* (see Figure 1(a)). This general model provides us with the possibility of formalising the design criteria of Section 3. We formalise the most important design criteria with the links to the design perspective as follows.

**L1 Topology Preservation.** Let *𝕃* and *𝕃'* be two layouts of the same transit network *N*. The layout *𝕃* preserves the topology of *𝕃'* if both have the same combinatorial embedding, i.e., this prohibits structural distortions such as modifying the circular edge orders around vertices or introducing additional edge crossings.

*Design Perspective:* No explicit correspondence, but correct topology is assumed as an obvious criterion in the design perspective. **L2 Straightness.** This measures, for a given transit line *t* € *T*, how many bends and inflection points it has in *𝕃*. For multilinear layouts, a high straightness implies use of as few bends as possible with preferably obtuse angles. For curvilinear layouts, uniform curvature and few inflection points are preferred.

*Design Perspective: simplification of trajectories*.

**L3 Distortion.** Let *𝕃* and *𝕃'* be two layouts of the same transit network *N*. The *degree of distortion* between *𝕃* and *𝕃'* measures the distance between the same station in *𝕃* and *𝕃'*.

*Design Perspective: global scale distortion, relative position of stations*.

**L4 Edge Length.** The edge length measures how uniform the lengths of the connection between consecutive stations are. *Design Perspective: even spacing of stations*.

**L5 Angular Resolution.** The angular resolution measures how uniformly incident edges are distributed around a vertex. *Design Perspective: layout types*.

**L6 Distance of Features.** Measures the distances between unrelated features. *Design Perspective: spatially‐seperated stations*.

Depending on the concrete problem setting, some of the listed properties are strictly enforced as hard constraints and some of them are optimised as soft constraints. In general, we can express most of the algorithmic results on creating the layout of transit maps as follows.

**Problem 1** (LAYOUT) Given a transit network *N* = (*S,C*), soft and hard constraints as well as the geographical layout *𝕃* of *N*, find a layout *𝕃'* in a given layout style that preserves the topology (L1) of *𝕃* and optimises the soft constraints subject to the hard constraints.

In order to express the soft constraints mathematically, cost functions for the different measures are introduced, which can be combined as a global objective function. Some soft constraints may contradict each other, for example, the straightness of the layout may be opposed to a low degree of distortion of the geographical layout. Hence, it is necessary to find a good compromise between them. A typical way is to linearly combine the individual cost functions and to balance them by weighted priorities.

#### 4.1.2. Algorithmic Aspect 2 – Labelling

**Figure 10:**
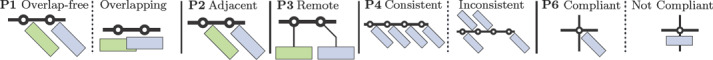
Illustration of labelling properties P1‐P4 and P6.

A *label* in a network map is a text, a symbol, or an image that describes a station. From an algorithmic point of view we abstract from this particular information and represent each label as rectangle, e.g., as its bounding box. Hence, for a transit network *N* = *(S, C)* we are given a set *L* of rectangles such that each rectangle *t* € *L* is a label of a station in *S*. For a layout *𝕃* of *N*, a *labelling ℙ* of *𝕃* places each label on *𝕄* by means of an affine transformation, i.e., it translates, scales and rotates each label *ℓ*, and places the result on the transit map *𝕄*. Based on the design criteria of Section 3 we distinguish the following basic properties of labellings; see also Figure 10.

**P1 Overlap‐free.** A labelling is *overlap‐free* if no two labels overlap.

**P2 Adjacent.** An *adjacent* labelling places each label *ℓ* € *L* with a fixed and small offset to its station in *𝕃*.

**P3 Remote.** A *remote* labelling places the label not necessarily close to the label's station in *𝕃*. In order to sustain the visual association between station and label, it additionally draws a connecting curve between both; this connecting curve is called a *leader*.

**P4 Consistent.** A *consistent* labelling places the labels of the stations along the same section of *N* on the same side.

**P5 Rotation.** The labels in a labelling are either horizontally, vertically or diagonally aligned.

**P6 Layout Compliant.** A labelling *ℙ complies with* a layout *𝕃* if none of the placed labels intersect a connection in *𝕃*.

Again, these properties can be enforced as hard constraints or optimised as soft constraints, which we formalise as follows.

**Problem 2 (LABELLING)** Given a layout *𝕃* of a transit network *N* = *(S, C)*, soft and hard constraints, find a labelling *ℙ* that optimises the soft constraints subject to the hard constraints.

Depending on the layout and the hard constraints, not all labels can be placed without creating overlaps with other labels or the network layout. Hence, many approaches also adapt the layout to create more free space for the labels (e.g., by stretching edges). In this case, a further soft constraint is that the layout is changed as little as possible. All of these approaches have in common that the resulting layout still has the same layout style, e.g., a too dense octolinear layout is transformed into another octolinear layout with more space for labels. In addition, we also consider approaches that consider Problem 1 and Problem 2 in an integrated way, i.e., these approaches create a labeled schematised layout in a single step.

**Problem 3 (Layout and Labelling)** Given a transit network *N* = *(S, C)*, soft and hard constraints for layouts and labellings as well as a geographical layout *𝕃*, find a layout *𝕃'* in a given layout style and a labelling *ℙ* such that *𝕃'* preserves the topology of *𝕃* and the soft constraints are optimised subject to the hard constraints.

#### 4.1.3. Algorithmic Aspect 3 – Transit Lines

**Figure 11:**
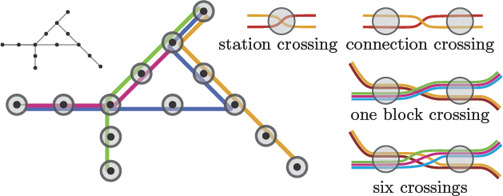
Each transit line is laid out such that it passes through the regions representing its stations.

Finally, we extend the model to drawing the transit lines of *T* along their connections in *𝕃* such that the total number of transit line crossings is minimal. We formalise this as follows. Each station *s* in *𝕃* is drawn as a region *r*_*s*_, e.g., a disk or square centred at *s* (see Figure 11). A curve c in *𝕄* represents a transit line *t* € *T* if it starts in the region of its first station, passes through all intermediate stations in the correct order and ends in the region of its last station in *𝕃*. Thus, the goal is to construct a set *𝕋* of curves in *𝕄* such that each transit line in *T* is represented by exactly one curve in *𝕋*; we call *𝕋* the *wiring* of *T* in *𝕃*.

**Problem 4 (Transit Line Wiring – Geometric Version)** Given a layout *𝕃* of a transit network *N* = *(S, C)* and a set *T* of transit lines, find a wiring *𝕋* with minimum number of crossings.

From a combinatorial point of view, routing transit lines can be formalised as an ordering problem. The curves representing the transit lines enter and leave the the stations in a certain order. Thus, for *N* we obtain an *ordering 𝕆* that describes for each vertex the order in which the transit lines enter and leave the station. Consider two transit lines passing through the same station *s*. They have a *station crossing* if the order in which they enter and leave *s* alternates (see Figure 11). Further, consider two transit lines that contain the same connection *e* € *C* between two stations *s*_1_ and *s*_2_. They have a *connection crossing* if they leave *s*_1_ and enter *s*_2_ in different orders. For common layouts (such as octolinear layouts) station crossings and connection crossings have a one‐to‐one correspondence with the crossings of the curves in *𝕋*. Hence, the combinatorial problem is to find an ordering *𝕆* of *T* such that the number of station and connection crossings is minimised. We observe that finding such an ordering is independent of the concrete layout as long as the topology is preserved.

**Problem 5 (Transit Line Wiring – Combinatorial Version)** Given a layout *𝕃* of a transit network *N* = *(S, C)* and a set *T* of transit lines, find an ordering *𝕆* of *T* such that the number of station and connection crossings is minimised.

### 4.2. Algorithmic Approaches: State of the Art

Table 3 gives an overview of the key properties of our references. In order to structure these, we have partitioned them into four groups. The first and second include those references that present algorithms for creating only layouts and only labellings, respectively. The third includes references that present algorithms for creating layouts and labellings in an integrated way or successively. The last group includes references that consider the wiring problem. In the following we discuss the four groups of references in this order.

#### 4.2.1. Algorithmic Layout Techniques

In this subsection we review approaches that consider only the layout problem (Problem 1) of creating transit maps. We group them with respect to the applied techniques.

**Line Simplification.** A core problem when creating schematic transit maps is the simplification of a path *P* such that the result is a *ℂ*‐oriented path *Q*, i.e., the orientation of each of its segments stems from the pre‐defined set *ℂ* of orientations. Neyer [Ney99] presented a dynamic programming approach that computes a *ℂ*‐oriented path *Q* that stays within an ε‐distance to *P*. While the approach takes the Fréchet distance into account, Merrick and Gudmundsson [MG07] used the Hausdorff distance slightly simplifying the problem by requiring that the simplified path needs to pass through vertices of *P* in the correct order. Dwyer et al. [DHM08] presented a simplification procedure that is based on least‐squares regression fitting octolinear or rectilinear paths to blocks of vertices. The procedure considers paths of the transit network independently, so that no design rules describing the interplay of transit lines are taken into account. In contrast, Barkoswky et al. [BLR00] considered multiple lines simultaneously using discrete curve evolution. Their iterative approach resolves the least relevant kinks by moving and removing vertices. While it preserves topology, the orientations of the edges are not restricted and the resulting layout does not satisfy any specific linearity criterion. Cabello et al. [CdBvK05] proposed an approach that schematises a road network by replacing paths between junctions with octolinear paths, each consisting of three segments. It returns a feasible solution (if one exists) or reports failure otherwise; no particular design criteria are optimised.

**Local Optimization.** Avelar and Müller [AM00] (see also [Ave07]) presented the first local optimisation method for creating schematisations of road networks. It iteratively moves vertices to nearby positions while preserving the topology of the network. The produced layouts only have few non‐octolinear edges. Ware et al. [WTAT06] presented a similar approach based on simulated annealing, which was extended by Anand et al. [AAWJ07]. Further, Ware and Richards [WR13] proposed an ant colony system in which the vertices are moved iteratively, but in parallel. Van Dijk and Lutz [vDL18] computed linear cartograms with prescribed edge length using least square optimisation. They utilised their algorithm to obtain a fast procedure for computing layouts of transit maps. However, their approach does not take any further design rules into account.

**Stroke‐based Methods.** Li and Dong [LD10] proposed a 3‐step algorithm for schematising road networks. Firstly, they decomposed the network into a set of strokes represented by paths. Secondly, they simplified the paths using a variant of the Douglas Peucker algorithm and projected the result on octolinear or rectilinear paths. Finally, they iteratively added the simplified paths to the layout, while preserving the topology by adapting the paths. Ti and Li [TL14] as well as Ti et al. [TLX15] presented a pre‐processing step for this approach that detects and enlarges congested areas with high feature density using a fish‐eye‐projection. Van Dijk et al. [vDvGH*14] used a stroke‐based approach, which represents each stroke by a circular arc, aiming for a curvilinear layout.

**Figure 12:**
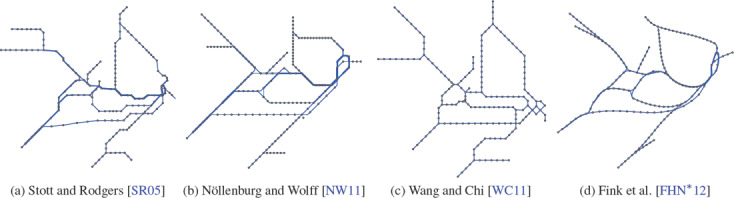
Layouts for the metro system of Sydney created by automated methods using (a) hill climbing (b) mixed integer linear programming (c) energy terms, and (d) force‐based methods. To emphasise the layouts, only their structures have been recreated from the original references, while the wiring of the transit lines and the labellings have been omitted. The thickness of the lines indicates the number of transit lines passing through the according edge.

**Force‐based Methods.** Fink et al. [FHN*12] used a force‐based method for drawing a layout based on Bézier curves instead of straight line segments; see Figure 12(d). The core idea is to change the position of the control points of the Bézier curves using forces. Chivers and Rodgers [CR14] presented a hybrid force‐based method that consists of two phases. Firstly, they used a spring embedder distributing the stations and creating a first rough layout. Secondly, they applied a magnetic force field in order to achieve octolinear edges. To improve the layout they used a gradual transition between both phases.

**Mixed Integer Linear Programming.** The mixed integer linear programming (MILP) formulation by Nöllenburg and Wolff [NW11] (see Section 4.2.3) was a starting point for several similar formulations. Oke and Siddiqui [OS15] improved the running time by relaxing constraints and dropping design rules in the objective. Nickel and Nöllenburg [NN19] generalised the formulation from octolinear layouts to multilinear layouts.

#### 4.2.2. Algorithmic Labelling Techniques

In this section we discuss approaches that only consider the labelling problem. The advantage of these generic labelling approaches is that they can be combined with many different layout algorithms. On the other hand, considering the layout and labelling problem separately bears the risk that the layout does not provide enough free space such that it must be distorted heavily. Hence, criteria that are optimised while creating the layout may become compromised. From the perspective of computational complexity, the problem is NP‐hard in several of its variants [GIM*01, NH18].

Wu et al. [WTLY11] presented a genetic algorithm that places textual labels and images on the metro map. They kept the layout fixed and used the free spaces of the map to place remote labels. At the core they used a genetic algorithm that evaluates the order of placement for the labels. Based on this they applied a greedy algorithm that places the labels while minimising the leader length.

Niedermann and Haunert [NH18] presented a procedure based on parametric search that systematically scales the edges of the map uniformly to acquire free spaces. Using this scaled layout they created for each station a set of label candidates. For the selection they considered dynamic programming, integer linear programming and a simple greedy approach.

Yoshida et al. [YMK*18] presented an iterative approach that places adjacent labels for each station from the inside to the outside of the map. In case that there is not enough free space, they scaled single edges preserving the shape and topology of the map. Takahashi et al. [TMK*19] extended this approach by restricting the aspect ratio of the regions enclosed by the network.

#### 4.2.3. Combined Algorithmic Layout and Labelling Techniques

Next we discuss approaches that consider both layout creation and label placement. We partition the results into those that strictly separate the layout and labelling problem and those that consider both problems in an integrated way.

**Successive Layout Creation and Labelling.** Hong et al. [HMdN06] used the force‐based paradigm for general graph layout algorithms. To that end, they proposed a topology‐preserving spring embedder that straightens transit lines and additionally uses magnetic forces that pull edges towards the closest octolinear orientation. In a second step, they created the labelling of the layout. They reduced the problem to finding a maximum independent set in a conflict graph based on label candidates for each station. The major drawback of their labelling approach is that stations may remain unlabeled.

Wang and Chi [WC11] considered focus‐and‐context maps in which a single travel route is highlighted and presented in the centre of the map. They formulated the layout problem based on weighted, squared energy terms that expressed the position of the vertices and the rotation of the edges. The labelling problem is solved in a second step again using a multi‐criteria energy function. In particular, it avoids occlusions between labels and prefers consistent labels on the same side of the metro line. The same approach can be used for creating layouts of the entire transit map; see Figure 12(c). Wang and Peng [WP16] used the same labelling approach for an interactive map editing system.

Wu et al. [WTLY12] presented an approach that creates a user‐specific metro map that shows a single travel route as a horizontal line in the centre of the map. They created this layout by modifying the MILP formulation introduced by Nöllenburg and Wolff [NW11]. They placed the pictorial labels on two sides of the boundary of the map, associated with leaders to their stations. Using a flow network they minimised the total leader length. Wu et al. [WPT*15] extended this approach to circular metro lines centred on the map and labels on all four sides of the map.

Wu et al. [WTH*13] introduced a procedure that creates a layout in three phases. First, they created an unlabeled schematic layout using a variation of the MILP of Nöllenburg and Wolff [NW11]. Afterwards, they enlarged the layout in order to acquire enough free space to place a pictorial remote label for each station. To that end, they again used a MILP approach to minimise leader length. Finally, they compacted the labeled layout so that labels were tightly contained within their respective faces.

**Integrated Layout Creation and Labelling.** Stott and Rodgers [SR05] and Stott et al. [SRMOW11] presented iterative multi‐criteria approaches based on hill climbing for creating the layout and labelling of a transit map in an integrated way; see Figure 12(a). The approach iteratively moves vertices and labels on a pre‐defined grid. Chivers and Rodgers [CR11] used the layout component of Stott et al. [SRMOW11] and introduced an approach for creating a labelling that greedily selects for each station one label out of eight candidates. In particular, they preferred the consistent placement of labels. Inspired by this approach, Chivers and Rodgers [CR13, CR15] conducted subsequent studies creating their layouts with hill climbing.

Nöllenburg and Wolff [NW11] introduced the first MILP formulation for creating transit maps; see Figure 12(b). It takes into account both the layout and labelling problems. The work was the starting point for several other approaches based on MILP formulations. As taking the labelling into account drastically increases the time for computing a solution, most of these considered the layout and labelling problem separately; see the previous section. Onda et al. [OMI18] used the method by Nöllenburg and Wolff at the core of their divide‐and‐conquer approach.

#### 4.2.4. Algorithmic Wiring Techniques

The transit line wiring problem has been studied mostly as a theoretical problem in the past, yet recently some algorithms have also been implemented. Most of the papers on the wiring problem consider the minimisation of connection crossings and explicitly forbid unnecessary station crossings, i.e., two lines may cross in a station only if they do not share an incident connection where that crossing could occur instead. This restriction is typically justified because station crossings are hidden underneath the station symbols and thus are more error‐prone in visual line tracking tasks.

The transit line wiring (also called *metro‐line crossing minimisation* (MLCM)) problem was identified by Benkert et al. [BNUW06], who presented an exact quadratic‐time dynamic programming algorithm for minimising connection crossings along a single edge in the presence of some terminating lines. Asquith et al. [AGM08] considered arbitrary planar graphs instead and presented a polynomial‐time algorithm for MLCM assuming that all lines terminate at a specified position at the periphery of the terminus station (MLCM with periphery assignment, MLCM‐PA); for the variant without given terminus assignment, but still requiring the periphery condition (MLCM‐P), they presented an ILP. Argyriou et al. [ABKS10] presented a faster algorithm for MLCM‐PA and showed that the MLCM‐P problem is N P‐hard, even for path networks. However, they also introduced another variant, where it is required that all transit lines in the network must terminate at leaf nodes of degree 1 in the underlying graph (MLCM‐T1). Nöllenburg [Nöl10] gave a faster algorithm for MLCM‐PA and MLCM‐T1 based on iterative line insertion on planar transit networks; finally, the running time to solve MLCM‐PA and MLCM‐T1 was reduced to linear time by Pupyrev et al. [PNBH16], who also implemented their algorithm. Fink and Pupyrev [FP13] extended the NP‐hardness of MLCM‐P to the unconstrained MLCM problem, even on a restricted class of tree networks. On the algorithmic side, they provided an approximation and a parameterized algorithm for MLCM‐P; if no transit line is a proper subline of another line, they showed that MLCM‐P can even be solved in polynomial time.

Fink et al. [FPW15] defined a variation of MLCM‐T1, in which the goal is not to minimise individual crossings, but the number of *block crossings* that combine all pairwise crossings of two bundles of transit lines into a single block crossing (see Figure 11), which can yield a visually cleaner appearance even if the number of individual crossings may be larger. They presented several algorithms and an NP‐hardness result for minimising block crossings.

Recently, Bast et al. [BBS19] presented a system for drawing geographical transit maps, which also includes a transit line wiring component for minimising station crossings. The wiring problem is solved either by engineering a sufficiently fast and exact ILP model or by alternative wiring heuristics based on hill climbing or simulated annealing.

### 4.3. Evaluation and Benchmarks

Evaluating and comparing solutions for automated layout, labelling, and wiring of transit maps is difficult for several reasons. Firstly, rating the aesthetic quality of the produced schematics depends on the same subjective measures as rating manually created designs. Since the design criteria and formal objectives to be optimised by an algorithm are never exactly the same when comparing two layout algorithms (even when they share many aspects), we also cannot resort to a purely quantitative evaluation in terms of objective function values. We have no proper, method‐ and map‐independent analytic measures for rating transit map quality, which ideally correlate not only to human aesthetic judgements, but also to task performance at these maps (see Section 5). Secondly, there is no widely accepted set of representative benchmark instances that is used for evaluating new layout solutions. Some network maps appear more frequently in evaluation sections than others (most notably, Sydney, London, Tokyo, Vienna, and Washington DC), but more systematic standard benchmark sets are needed. Therefore, most of the practical papers present a collection of sample layouts produced by their method, sometimes in comparison to official maps from the transport authorities or layouts found in previous work. The analysis of these new layouts is then mostly anecdotal, discussing pros, cons, and the main differences to competing maps. Some of the papers also report results from a formal user or expert study; see Table 3, M5.2. Therefore, in our comparison here, we do not attempt to provide a complete ranking of the methods in the literature. Rather we carefully inspected the example layouts provided in each paper and judged whether the commonly expected properties of a schematic transit map as discussed in Sections 4.1 are *mostly satisfied, somewhat satisfied*, or *barely satisfied*.

A second aspect for evaluating implemented algorithmic approaches for transit map layout is their actual computational performance. Some algorithms are very fast and provide schematic maps in less than 1 or 2 seconds, which allows their use in an interactive setting providing basically instantaneous output. Other algorithms have some lag from several seconds to a few minutes before returning the output. Such algorithms may still be well‐suited to assist graphic designers and cartographers in a continuous map creation workflow with acceptable waiting times. Finally, some of the methods require even longer computation times up to several hours or more, meaning that layouts can be computed overnight. Even though these three performance groups are coarsely defined, it can still be difficult to put a method into one of them precisely. This is due to the fact that the running times reported in the literature originate from vastly different hardware systems, from desktop computers to powerful servers, and having more than 20 years of age difference. Secondly, the instance complexity between a smaller network like Vienna and a large one like Tokyo differs drastically; it is very difficult to predict running times on large and complex instances based on measurements on small and simple instances.

In Table 4 we present all those references discussed in this section, which provide sample layouts and running times. Each layout method is assigned to a group regarding the level of design criteria satisfaction and a group regarding computational performance. Algorithms for which no performance data are reported in the papers are listed in the top row. References that are in one or even both of the highest scoring groups represent algorithms that satisfy the respective aspect(s) well. This does not necessarily mean that a method is able to generate schematic transit maps perfectly or in the same quality as a human designer would do. For instance, methods that can solve the layout or wiring problem very well do not necessarily integrate proper station labelling or some of the more global aesthetics such as symmetries, harmony, or balance.

**Table 4:** Ratings for the approaches found in literature with respect to satisfaction of typical design criteria (barely, somewhat, mostly) and performance (unspecified, slow (> 2 min), lagged (≤ 2 min), instantaneous (≤ 2 sec)).

		Design Criteria Satisfaction		
		barely	somewhat	mostly
Performance	unspec.	[AM00], [BLR00], [LD10], [TL14], [TLX15]		
	slow		[CR11], [OMI18], [SRMOW11], [SR05], [WTLY12], [WTLY11]	[NW11], [WTH*13], [OS15], [NN19]
	lagged	[HMdN06], [AAWJ07], [WTAT06]	[CR13], [CR15], [TMK*19], [WPT*15], [vDvGH*14], [WR13], [YMK*18], [NH18]	
	instant.	[DHM08], [MG07]	[WC11], [vDL18]	[WP16], [CR14], [PNBH16], [BBS19]

One observation that can be made from Table 4 is the improvement of algorithmic techniques over time (see Figure 12 for some sample layouts). For instance, algorithms in the lowest design criteria satisfaction bracket all belong to the earliest approaches pioneering the automation of metro map layout. The more recent techniques are grouped more towards higher levels of criteria satisfaction and better performance and are mostly found on or below the diagonal from bottom left to top right.

## 5. Human Perspective

Research in the human perspective category has the objective of attempting to understand transit maps from the point of view of the user, including how designs are perceived and interpreted, along with the strategies that people apply for route planning. Hence, it is intended that the effects of design on usability will be understood, so that it is possible to identify and subsequently specify the criteria necessary for efficient error‐free performance.

One strategy for researchers is to extrapolate from general behavioural sciences findings to the particular case of transit maps [Ros11]. In many cases, it can be difficult to identify precise specifications for effective design, but findings can offer tools to critique existing maps [Rob17]. It is advisable for researchers to attempt to validate their extrapolations via empirical testing of actual transit maps.

Another strategy is to obtain data from the use of transit maps themselves. Where available, real route choices can be investigated using regression analyses with respect to the map design features [Guo11]. Alternatively, designs may be tested in experimental studies, in which users are asked to perform various tasks and qualitative or quantitative aspects of their performance are measured. From these, the effects of design on performance can be identified and evaluated, and prescriptions for good practice formulated.

Table 5 gives an overview of 26 papers that contribute to the human perspective. There are two earlier survey articles which give an overview of usability testing [Rob14a, Gri19] along with a more detailed review of the effects of topographical distortion on usability [For14]. In conjunction with the current analysis, these give a comprehensive overview of past research, highlighting the topics that have received the most focus, along with the generalisable design principles that can be identified from them.

**Table 5:** Papers investigating the effects of various aspects of schematic map design on the measured behaviour of the user, categorised by (H1) the research topics investigated – primary (o) and secondary (x); (H2) the independent/regression variables that were analysed statistically (A) or investigated on an informal basis (I); and (H3) the tasks/measures of performance that were recorded in order to evaluate the designs; and (H4) the studies with implications for schematic map design (see 5.4 for explanation of codes).

	Human Perspective Publications																										Σ 26
	[Bar80]	[Bur18]	[BD84]	[BDO76]	[BKW14]	[BRB97]	[BRB*14]	[EAM77]	[GGBW17]	[GHG79]	[Guo11]	[GZW*17]	[Hoc09]	[LRR18]	[MG19]	[NOK*17]	[Ros11]	[RGL17]	[RGM12]	[RMdG11]	[RNC16]	[RNL*13]	[RR16]	[RV16]	[Ver08]	[Xu17]	
**H1 Primary and secondary research issues**																											
H1.1 Effects of macro layout (global map, e.g., design rules)			o															X			o	o		o			5
H1.2 Effects of macro formatting (e.g., stroke thickness)																	X										1
H1.3 Effects of micro layout (configuration at specific locations)											X	o	X	X	X				X	X			o			o	9
H1.4 Effects of micro features (e.g., configurations of transfers)				X																							1
H1.5 Effects of colour‐coding			X							o				o		X											4
H1.6 Effects of topographical distortion			X			o					X														o		4
H1.7 Effects of supplementary information (e.g., surface details)	o	X						X		o			X														5
H1.8 Identification of sources of difficulty in using metro maps							o																				1
H1.9 Identification of visual scanning behaviour for metro maps		o							o																		2
H1.10 Identification of route planning processes for metro maps					o				o							o											3
H1.11 Effects of information format and complexity/level of detail								o																			1
H1.12 Factors that influence route choice											o	o	o		o				o	o		X	o			X	9
H1.13 Effects of city expertise on route choice																										o	1
H1.14 Factors influencing early visual stages of map perception																	o										1
H1.15 Evaluation of fitness of existing design(s)			o	o				o			o	X		X			o	X				o	o		X	X	12
H1.16 Objective versus subjective measures of performance																		o			X	X	X	o			5
**H2 Variables analysed/investigated informally**																											
H2.1 Macro‐layout			A															A			A	A		A			5
H2.2 Micro‐layouts											A	A	A	A	A				A	A		I	A			A	10
H2.3 Micro‐features: configuration of transfer stations				I							A																2
H2.4 Level of city expertise			A															I								A	3
H2.5 Cross‐cultural factors																			A								1
H2.6 Route complexity necessary to complete journey	A						A		A							A											4
H2.7 Network size/complexity		I					A									A											3
H2.8 Information format/complexity/level of detail								A																			1
H2.9 Colour‐coding		A	I						A							A				A		A					5
H2.10 Presence of topographical features	A							A		A																	3
H2.11 Supplementary information: services, facilities, etc		I											A														2
H2.12 Thickness of line bundles																	I										1
H2.13 No variables discussed or analysed					I	I																			I		3
**H3 Tasks and Measures**																											
H3.1 Route planning time	X						X		X	X						X		X			X	X	X	x			10
H3.2 Route planning errors (i.e., non‐valid routes)			X	X						X					X								X				5
H3.3 Route choice											X	X	X		X				X	X		X	X			X	9
H3.4 Estimated route efficiency (e.g., no of transfers, directness)			X	X					X		X					X		X			X	X	X	x			10
H3.5 Direct route availability verification (time and errors)														X									X				2
H3.6 Station finding time	X						X		X														X				4
H3.7 Gaze direction/duration		X			X		X		X							X											5
H3.8 Estimated station position (show on map)						X																					1
H3.9 Logistical details of planned routes (errors)								X		X																	2
H3.10 User evaluations (e.g., map choice, rating questionnaires)										X								X			X	X	X	X			6
H3.11 Draw a map showing key features of a city																									X		1
H3.12 Verbal protocols of reasoning/planning strategies						X																					1
H3.13 Computer simulation																	X										1
**H4 Findings with implications for schematic map design**																											
H4.1 Visual field (DR1)																	X										1
H4.2 Micro‐layout/route choice (DR2)											X	X			X				X	X		X	X			X	8
H4.3 Validity of user preferences (DR3)																		X				X	X	X			4
H4.4 Line colour coding/route tracking (FR1)														X													1
H4.5 Macro‐layout/octolinearity = gold standard (FR2)																		X				X		X			3
H4.6 Micro‐layout/route tracking (FR3)				X										X													2
H4.7 User‐understanding of topographical distortion (FR4)						X					X																2
H4.8 Need for usability measures with practical utility (MR1)																					X						1
H4.9 Need for measures of network learning (MR2)																		X			X						2

### 5.1. Variables in Usability Testing

**Figure 13:**
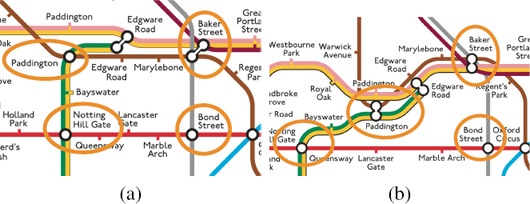
Octolinear maps of London, (a) optimised for simple line trajectories, similar to the official Underground map (b) is topographically correct. For (a) the two alternative routes from Paddington to Bond Street appear equally viable. In a regression study of real route choice data, map distance was more predictive of journey choice than topographical distance [Guo11]: 40% of passengers took the less efficient route in this specific example.

**Figure 14:**
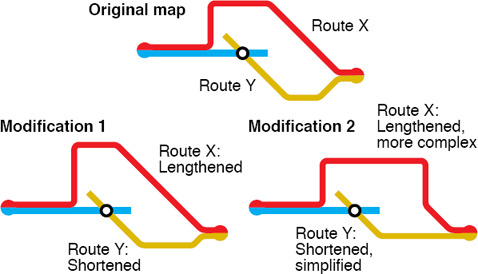
Examples of experimental manipulations of layout to investigate, via laboratory studies, its effects on route choice (as per [GZW*17, Xu17]). Route X is more popular because it offers a one‐seat ride, and hence might be overcrowded.

The variable most frequently investigated is the effect of micro layout. Different configurations can be identified [Guo11], categorised [LRR18] or manipulated [GZW*17, Xu17] and the consequences of these investigated – usually for route choice (Figures 13, 14). Colour‐coding has also frequently been investigated, but mostly only comparing this with no coding at all. The one exception [LRR18] investigated the effects of colour‐coding individual routes (route colour‐coding) versus using identical colours for routes on a common trunk line (trunk colour coding).

Macro layout has been investigated in a number of studies, in which the entire network is mapped using different design rules, e.g., octolinear versus curvilinear [BD84, RNL*13, RV16], octolinear versus curvilinear versus multilinear [RGL17] and octolinear versus concentric circles and spokes [RNC16]. There can be difficulties in interpreting such studies, although the question of which design rules result in the most effective designs generally, or for specific networks, is an important one.

Individual difference variables have been investigated comparatively rarely, so far being confined to city expertise [BD84, RGL17, Xu17] and nationality – British versus Chilean [RGM12]. Also rare, considering the variability of applications in practice, is investigation of the effect of supplementary details on route planning performance, such as topographical features and information about services and facilities.

The three most frequent measures of map usability are route choice, route planning time – the time necessary to identify a route between two specified stations – and route efficiency – based on time/number of transfers/inconvenience estimates for chosen routes. All of these are straightforward to implement and have yielded reasonable effect sizes in the literature. In general, studies of micro layout tend to investigate route choice, whereas studies of macro layout tend to investigate route planning time.

Other measures have been investigated less frequently for good reason. (1) Route planning errors, in which illegal routes are formulated (e.g., requiring a non‐existent transfer between lines) are relatively rare for a competently designed map. (2) A number of studies have investigated gaze direction/duration, but so far the conclusions identified struggle to justify the extra effort necessary to implement the methodology. (3) Station finding time (locate a named station on the map) has a considerable chance element, and therefore results in high levels of variability between subjects, reducing statistical power.

A number of measures have been investigated infrequently but deserve wider attention. (1) *direct route verification tasks* – Is a direct route available between station *A* and station *B*? – which has yielded significant differences between designs both for complex [LRR18] and simple networks [RR16]. (2) two studies asked subjects to draw/place missing information, identifying their mental models for city structure [Ver08] and for the topographical literalness of a schematised map [BRB97]. (3) a minority of studies attempted to ascertain users’ opinions about the usability of designs that were being tested, e.g., by asking subjects to identify preferred designs when given a range to choose between, or by rating individual maps according to a number of criteria associated with usability, such as ease of following lines, discriminating lines, and negotiating interchanges. (4) one study implemented a computer model of human peripheral vision, and applied this to different designs of maps, generating a dramatic visualisation of differences in the degradation of designs away from visual focus [Ros11].

### 5.2. Research Topics and Key Findings

The focus of this section will be on cases where multiple papers have addressed the same research topic: in combination they form clear, well‐corroborated findings that offer important insights for people with an interest in transit map design. A number of other individual papers will also be highlighted which offer clear and/or potentially interesting findings worthy of further research.

#### 5.2.1. Effects of Micro Layout on Route Choice

**Figure 15:**
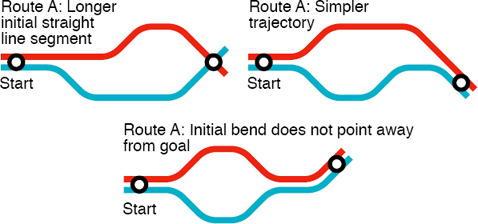
Summary of key findings on the relationship between layout and route preference. In each case Route A is preferred.

A number of studies, adopting broadly comparable methodologies, have investigated this topic [Guo11, MG19, RGM12, RMdG11, RNL*13, RR16, Xu17, GZW*17]. Of these, regression studies quantify various aspects of layout and perform regression analyses to account for route choices [Guo11]. Experimental studies compare route choices between maps with different designs, ascertained either by asking subjects to generate their own routes [RR16] or else by offering multiple pre‐determined routes and asking them to choose between these [GZW*17]. Together, these studies provide a clear picture of how micro layout affects route choice. Overall, the preference is for options with the fewest interchanges and with the shortest travel distance – implied by line segments length on the map – [Guo11] with the following caveats (Figure 15):

- Complexity. A route with fewer bends will be preferred to a route with more bends [RNL*13].
- Directness of initial segment. A route whose initial segment points directly towards a destination will be preferred over routes whose initial segments point away [RMdG11].
- Interchange postponement. A route will be preferred with a later interchange than an earlier one, even if this results in a less direct result overall [RR16].

A further study [GZW*17] investigated a redesign of the Washington, DC metro map with the intention of shifting preferences from an overcrowded route, with zero interchanges, to a target, less crowded route which required one interchange. The greatest shift was for the map which lengthened the overcrowded route the most, making it appear even less direct. However, a further study [Xu17] found that this was only effective for people less familiar with the city. For people more familiar, the greatest shift was for the map in which the bends on the target route were straightened. These findings are easily explained – for less familiar people, the map that made the overcrowded route look implausible deterred them from choosing it. For more familiar people, they already knew that this was a plausible route, and so the most effective design enticed them away from it by making the target route more attractive. Hence, although the micro layout factors that influence route choice are generally understood, putting these into practice, e.g., to relieve overcrowding, may require knowledge of which types of user should be targeted, and hence an understanding of the mediation effects of relevant individual difference variables.

#### 5.2.2. Effects of Macro Layout

Three studies have investigated the effects of macro layout by comparing maps of the same network (e.g., Paris, Berlin, London) depicted using different design rules [RGL17, RNL*13, RV16]. Map effectiveness was measured by comparing route planning times, and the one constant finding across all studies is a failure to support the conjecture that *octolinearity* = *a schematisation gold standard*. This reflects the widely held belief amongst researchers, designers and the general public (see Section 5.2.3) that octolinearity will always provide the most effective layout from a usability perspective. In every single case, for the six experiments reported in these three studies, octolinear designs either yielded performance no better than alternatives, or significantly worse.

This finding is important considering the suggestion [Rob12] that different networks are structured in different ways, and hence may be more or less compatible with different design rules with respect to optimisation according to criteria for effective design, such as simple line trajectories and minimised topographical distortion. If the use of octolinearity is no guarantee of the most effective design, then this opens the door to investigating the use of different design rules for different networks.

#### 5.2.3. Subjective Evaluations of Usability

Five recent studies have investigated user ratings of designs, using various measures that have proven to be correlated and internally consistent [RGL17, RNC16, RNL*13, RR16, RV16]. These include map choice/preference rank ordering, and scores obtained from rating questionnaires. The most persistent, surprising finding is zero association between subjective evaluations versus objective measures of effectiveness. This indicates that researchers’ conceptualisations of usability are effectively orthogonal to those of users, which is an important finding in its own right: map acceptability, or even desirability, is a necessary objective for designers, otherwise there is a risk that users (and transport professionals) will simply reject maps and turn to journey planning software instead.

The most comprehensive investigation [RGL17] found that subjects displayed a clear, strong octolinearity preference: such maps tended to be rated far more highly for usability than multilinear and curvilinear equivalents. There was also a clear, but slightly less strong simplicity preference: maps with simpler line trajectories tended to be rated more highly than those with the same design rules but more complicated trajectories. The preferences of people whose ratings showed the greatest levels of internal consistency were mutually exclusive: almost without exception either for octolinearity, or simplicity. Hence, individual differences in map evaluations are substantial.

The maps in this study depicted the London Underground network, and it is possible that the octolinearity preference resulted from familiarity with the London Underground map. However, there were no substantive differences in ratings comparing British, American, and German respondents. Also, subjects were asked to rate the maps separately for usability and attractiveness, and scores showed a clear dissociation. Octolinear maps were rated highly for both criteria, but multilinear maps as being more usable than curvilinear ones, and curvilinear maps were rated as being more attractive than multilinear ones. Hence, processes underlying usability judgments by the general public seem to be more complex than mere familiarity or first impressions of attractiveness.

The responses of people who claimed to have a relevant professional background (design or transport), or some other interest in schematic map usability, were almost identical to those from people who did not have these backgrounds. Hence there is the interesting but disturbing implication that assessment of usability of maps by people with at least some expertise in this domain is indistinguishable from the general public.

#### 5.2.4. Other Studies

**Figure 16:**
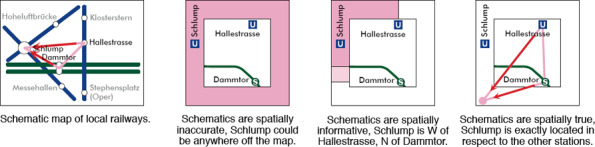
In a study investigating topographical understanding [BRB97] subjects were given a schematic of local railways and a topographical map cropped so that Schlump was missing. Asking subjects to indicate its likely location yielded considerable individual differences.

Two studies have nicely shown the importance of understanding the effects of schematic map topographical distortion on the user. People's mental models of the structure of London's streets, landmarks and rivers tend to be distorted in such a way that matches the spatial layout of the London Underground map [Ver08]. In another study [BRB97], people were given a schematised railway map of an unfamiliar city and a topographical street map. They were asked to predict the location of a target station that was beyond the area shown on the topographical map, and many people used the exact spatial relationships of the schematic to infer this (see Figure 16). Both studies showed considerable individual differences in people's tendency to treat the layout of a schematic map as being literally true, but nonetheless the findings indicate that designers need to take this issue into account.

**Figure 17:**
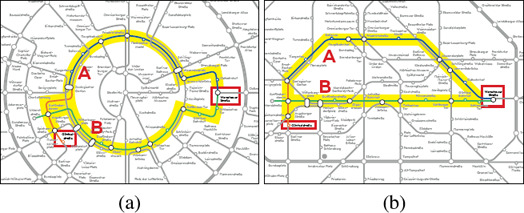
A concentric‐circles‐and‐spokes map (a) of the Berlin U‐Bahn and S‐Bahn networks proved to be both slow for planning routes and unpopular with subjects, compared with an octolinear alternative (b) [RNC16].

Most investigations of global layout have failed to demonstrate any usability superiority for octolinearity (Section 5.2.2). For the one exception [RNC16] a conventional Berlin octolinear map was compared with one based on concentric circles and spokes radiating from the centre. Such maps generate considerable Internet comment and media attention, and it can be argued that they exhibit high degrees of orderliness and organisation. However, in actual testing the concentric circles map yielded *both* adverse route planning times, *and* poor user ratings. The reason for low ratings appears to be that their structure made the desirability of route options difficult to compare (see Figure 17). These findings highlight the importance of evaluating novel designs via usability testing, irrespective of their informal receptions. Also, although user evaluations are generally uncorrelated with objective measures (as was the case in this study) these can nonetheless yield important insights. In a few cases, the structure of actual cities is based upon concentric circles and spokes, such as Cologne and Amsterdam, and for these the advantages of a concentric circles and spokes map matching the structure of a city might outweigh possible navigational difficulties [NR18].

**Figure 18:**
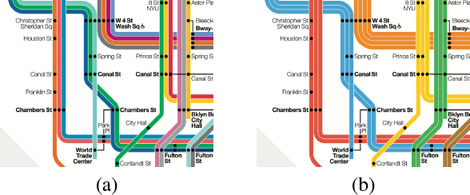
Maps from an investigation of colour coding: (a) route versus (b) trunk [LRR18]. Subjects were asked “is there a direct route from A to B, yes or no” and error rates were generally better with route colour coding.

Colour coding of metro lines differs substantially from network to network, most obviously with some adopting route colour coding and others adopting trunk colour coding. The choice of which system to adopt seems to depend on national traditions rather than any basis in usability testing. Initial studies [LRR18] have shown that route colour coding yielded the best performance in a *direct route verification task*, i.e., one in which it was necessary to track individual lines and pay attention to the stops *en route* (Figure 18). This study combined an investigation of colour coding with micro layouts, identifying a number of *navigational hazards:* particular layouts where the difficulty of accurately tracking routes was greatest. Tracking lines for long distances proved unchallenging for either colour coding, even when lines passed under others, but trunk colour coding was challenging in situations where lines ceased to run in parallel. The map used in this study was a variation of the famous 1972 Vignelli design [LO12]. The level of errors for this task was surprisingly high, approaching 40% in some conditions, perhaps vindicating earlier complaints about its usability [BDO76].

**Figure 19:**
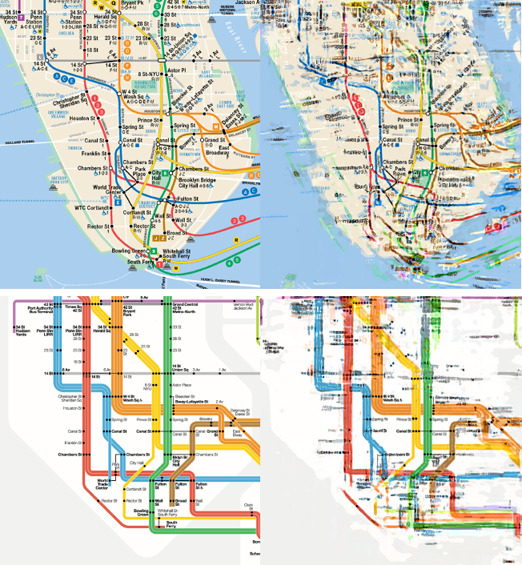
Computer modelling by Ruth Rosenholz of peripheral vision information attenuation for two different New York City Subway maps: Official MTA topographical design (top), MTA weekender map (bottom). In each case the visual focus is Canal Street. Thin lines with complex trajectories are particularly vulnerable. For example, on the topographical map, the lines almost disintegrate as they head northwards through Manhattan [Ros11].

Considerable research exists on the properties of the human visual system, but little of this has filtered back to transit map design. One exception [Ros11] investigated the consequences of signal attenuation in peripheral vision, which preserves structure and movement at the expense of detail. Computer simulations of this demonstrated, via dramatic visualisations, the effects of this for different macro formattings. Maps with disorganised thin lines and cluttered with supplementary information were particularly vulnerable, indicating that peripheral vision is less likely to be able to supply information useful for navigating these (see Figure 19). This study highlights the potential of creating computer models of user perceptual and cognitive processes as a means to evaluate maps without the need for extensive usability testing.

### 5.3. Issues in Usability Testing

Usability studies can be demanding of resources – hundreds of subjects have been tested in many cases [GZW*17, LRR18, RGL17, Xu17] – and there may be limits to the sorts of issues that studies can resolve (see Section 6.3) along with limits to the generalisability of their findings (see below). There is also a possibility that researchers focus attention on issues that are easy (a) to operationalise as independent variables and (b) to measure as dependent variables.

#### 5.3.1. Missing Topics and Measures

The most obvious complaint that can be levelled against many usability studies is that they are not investigating real journeys. Much of the research involves paid volunteers, e.g., planning multiple routes between origin‐destination station pairs, with no intention of actually implementing these. Hence, the behaviour patterns identified in such studies may be artifactual rather than representative. In defence of usability studies, many of these have identified differences between maps for errors made while tracking routes, or for the time taken to plan these [LRR18, RNL*13]. As such, adverse performance at these measures is assumed to be indicative of general difficulties in interpreting and using such designs. The reasonable assumption is that the most difficult maps in laboratory settings will also prove to be the most difficult ones to use in reality.

For studies in which the prime measurement is route choice, there is a possibility that the inefficient routes chosen in laboratory settings would not have manifested themselves in the context of real settings – where inefficient routes have actual time costs. It should be noted that substantial proportions of inefficient routes have been observed when datasets of real journeys have been analysed [Guo11]. Furthermore, the biases found in laboratory settings can be substantial. For example, Roberts and Rose [RR16] observed that subjects proposed four times as many inefficient routes for one prototype design versus alternatives and noted that (p. 458) “it would be a very brave transportation authority, or operator, indeed, that chose to disregard any such warning signals identified in this way”. Some caution in interpreting laboratory studies is wise, and validation of findings desirable, but a blanket rejection is not warranted.

In terms of future focus for researchers, the effects of macro and micro layout have been well‐investigated, but very few studies have sought to understand the effects of topographical distortion on usability. The permissibility of topographical distortion is a fundamental issue in the creation of schematic maps, subject to considerable disagreement between practitioners and users, and is in conflict with the criterion for simplified line trajectories. Together, these elevate the lack of research on this topic to a challenge that needs to be addressed (see Challenge 3, Section 7). There are many other aspects of design that could benefit from evidence‐based input but for which current research is sparse, such as optimal macro formatting (line thicknesses, types of station and interchange symbols) and micro features (e.g., interchange station layouts) and whether prescriptions for design should be modified depending on whether the intended audience is expert or novice users.

Route choice, route verification errors, and route planning time have all proved to be straightforward and useful variables to measure. User assessments of designs can also be identified easily, with the interesting caveat that attractiveness of a design is perceived to be different from usability. Harder to measure, but perhaps crucial to the future of maps, is people's learning of a network as a positive, yet incidental, consequence of using one to plan routes. This is alleged to give map‐based navigation an advantage over computerised alternatives [RGL17] and it is also suggested that trunk colour coding might have an advantage over route colour coding in this respect [Rob12]. The challenge is to devise and validate measures of network learning that are not subject to floor effects for complex networks, such as the New York City Subway.

#### 5.3.2. Specific Evaluations Versus General Principles

**Figure 20:**
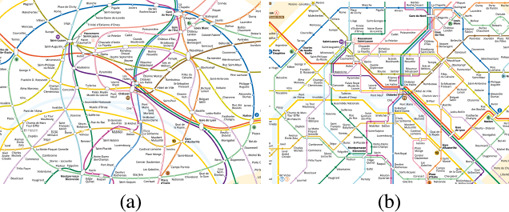
A curvilinear map of the Paris Metro (a) has been shown to be faster for route planning than the official octolinear design (b) in several experiments [RNL*13, RGL17].

Usability testing is powerful in terms of identifying the most and least effective designs from amongst a set of prototypes [RR16]. However, translating differences between specific designs into generalised design prescriptions is harder. A good example of this is comparisons between the official octolinear Paris Metro map and a curvilinear alternative [RNL*13, RGL17]. Despite the consistent faster route planning time for the curvilinear map, it would have been incorrect to have concluded that curvilinear designs should be preferred, even for the Paris Metro. This is because the maps varied by their design priorities as well as their design rules, with simple line trajectories being a priority for the curvilinear map but not for the octolinear one (see Figure 20). Hence, a weaker conclusion was put forward [RNL*13], i.e., that there had been a refutation of the conjecture that *octolinearity* = *a schematisation gold standard*. These issues have been discussed in detail [Rob14a] and it has been noted that even with maps designed with utmost care, it is almost impossible to ensure that they are perfectly matched across all possible variables except the ones that are intended to be manipulated. Hence, researchers need to exercise caution in generalising from their findings.

#### 5.3.3. The Usability Gap

Despite the importance of the acceptability of new designs, only a minority of studies have solicited user opinions or preferences. The exceptions [RGL17, RNC16, RNL*13, RR16, RV16] have all identified a persistent dissociation between objectively measured performance and subjectively assessed usability. Hence, users often prefer ineffective designs and reject effective ones, and this phe‐nomenonhas been named the *usability gap* [Rob19c]. It is possible, but inadvisable, to dismiss the disagreement as poor judgement by subjects on the basis of their inability to monitor their own performance. It is important that measures of route planning performance are directly relevant to the adverse consequences of poor design: they should have *practical utility*. Route planning errors (choosing impossible routes) have obvious practical utility. The status of route planning time is less obvious: designs might differ by tens of seconds, but this time is small compared with the cost of implementing an inefficient route, which might be tens of minutes. The low correlations between user‐ratings of maps versus measures with low practical utility, such as route planning time, indicate that it would be premature to dismiss the usability gap as a reflection of poor judgement by users.

### 5.4. Summary of Key Findings of the Human Perspective

Research on topics related to the human perspective is expanding, with a number of findings that have direct relevance (DR) for other perspectives, or else indicate that further research (FR) will pay dividends. However, various issues indicate that methodological refinement (MR) is also necessary.

**DR1.** Research into visual perception indicates that certain design parameters should be adopted in order to ensure that maximal layout information is available throughout the visual field.

**FR1.** Line colour‐coding conventions differ widely internationally, and initial findings suggest that the system used can have direct consequences for performance, such as line tracking errors.

**FR2.** Studies have cast doubt on the *octolinearity* = *gold standard* conjecture, clearing the way for a more flexible approach to schematisation, adopting design rules to match network structure.

**FR3.** Micro‐layout research has identified line configurations that are particularly likely to lead to errors in tracking routes.

**DR2.** Much research has focused on the effects of micro‐layout on route choice, with clear implications for designers who, for example, might wish to encourage people to alter their choices.

**FR4.** Differences in user‐understanding of topographical distortion indicates a need for caution in applying this to schematisations, especially where this might lead to inefficient route choices.

**DR3.** Several studies highlight that designs that are popular with users are not necessarily the ones that are the most usable, hence user‐preferences should not be relied on when adopting designs.

**MR1.** The dissociation between objective measures of performance versus subjective evaluations indicates that measures of usability with high practical utility are most desirable.

**MR2.** Researchers have hypothesised that schematic maps, both in general, and designed in certain ways, facilitate network learning, and validated measures of this are required.

## 6. Fusions of Multiple Perspectives

Historically, the three perspectives have typically worked in isolation, but there is a growing realisation that a multidisciplinary approach is necessary to advance further. The following discussions identify potential interrelationships between perspectives, and opportunities for mutual strengthening, as well as possible difficulties in interfacing between them.

### 6.1. Between Design and Machine Perspectives

The literature described in the design perspective (Section 3) provides a summary of how illustrators create transit maps through the combination of universal design principles as well as their preferences and experience, broadly speaking *design sense*. Computer scientists benefit from the results of design and user studies, which facilitate the synthesis of designers’ ideas into a set of principles and assist them in transforming these into a set of mathematical expressions that can be understood by machines. However, this procedure may lose the aesthetic essence due to the limited capability of the machine or the programmer. One example is the expansion of the centre of a transit map to avoid dense information at certain regions [HMdN06, SRMOW11, NW11]. If we formulate this by only incorporating equally spaced edges, this could lead to a result that only partially fulfils the principle due to the gap between designer's and machine's conceptualisation. The goal of the machine perspective (Section 4) is to minimise the gap so that a machine can reproduce very similar results as can be created by a designer.

**Contribution of the design perspective to the machine perspective.** Machine‐generated maps have proved their quality to a certain degree [WNTN19]. However, to fully satisfy the visual quality expected from a transit map, to tackle only a subset of criteria in the design perspective is still insufficient. This is because the human solution is, in general, more holistic, and some design choices made by an illustrator cannot yet be quantified. The lack of automatic evaluation methodology also limits the development of such an intelligent approach due to uncertain goals. Most of the pioneering approaches [HMdN06, SRMOW11, NW11] only allow a parametrised set of predefined variables in the algorithm, but they cannot adaptively prioritise criteria [OS15]. This gives another level of complexity on top of layout or labelling problems, each of which already have high computational complexity (see Table 4). For this reason, one can consider the results generated by a machine as a base map for refinement. Some techniques incorporate a human‐in‐the‐loop editing process [WC11, WP16], but a fully controllable interactive approach is still an unfulfilled goal [WNTN19]. Searching for a compromise between computer performance and visual quality is an ongoing research issue.

**Contribution of the machine perspective to the design perspective.** Empirically, we believe that the design perspective has a stronger impact towards the machine perspective, due to the reasons that the present approaches are mostly built on top of the selected design criteria. An analysis has been performed to investigate the correlation between incorporated criteria, corresponding visual quality of machine‐generated transit maps, and system interactivity [WNTN19]. The formal machine perspective pushed designers towards more precise principles, i.e., rather than saying “this looks better”, they are urged to give reasons that can be defined, such as “it has fewer bends” or “the pattern of lines is more symmetric”, etc. Since computers facilitate designers to perform rapid prototyping, it becomes easier to try maps with different design rules, as well as of novel visualisation ideas and concepts. This mutual communication between design and machine perspective allows scientists to establish relationships between the design space and the machine space, so that one can identify and utilise the design criteria and their corresponding formulation, to assist evolution in both perspectives.

### 6.2. Between Machine and Human Perspectives

Research taking the machine perspective mainly focuses on the development of methods for creating transit maps automatically (see Section 4). One of its major challenges is then assessing the quality of the produced map. On the other hand, the strength of research taking the human perspective is the evaluation of maps, while it has a high demand for transit maps that can be used in empirical studies (see Section 5).

**Contribution of the machine perspective to the human perspective.** Investigating the usability of transit maps requires a variety of examples possessing similar layout properties. Depending on the extent and goal of the planned study, creating these transit maps manually is complicated or just not possible. Firstly, even creating a single transit map is a challenging task that requires experienced designers. Thus, conducting a study on a variety of maps easily becomes too time‐consuming. Automated methods promise to mitigate this obstacle to widespread user studies. Once an algorithm has been developed for creating a layout style, it is only a question of computation time to create a variety of instances. This of course requires that the development of automatic methods has reached a level that can compete with manually created maps. Secondly, in order to obtain meaningful studies systematically testing hypotheses, the considered layouts should possess controlled properties; see Section 5.3.2. For example, when investigating the impact of bends on the usability of a transit map, the number of bends should be varied while other properties, such as the drawing area, should be kept fixed. For this purpose of varying single properties we especially deem approaches based on mixed integer linear programming (e.g., [NW11]) to be the current best choice. Their advantages are two‐fold. First, they yield layouts of high quality including labelling and the wiring of transit lines [BBS19]. Second, such algorithms yield mathematically optimal solutions for an objective that is subject to a set of hard constraints. The soft constraints constituting the objective and the hard constraints can both be easily manipulated and controlled. We note that for creating stimuli, heuristics are not adequate as they do not give guarantees on the properties of the produced layout, thus risking to confound the results. Automated methods also open up new research fields for the human perspective. For example, with the ubiquity of mobile devices such as smart phones, customised and dynamic maps became part of our daily life. This development has driven the introduction of new concepts for transit maps such as focus+context maps [WC11] and travel‐route‐centred metro maps [WTLY12, WPT*15]. These concepts are inseparable from automated methods. From the human perspective their usability is generally not well understood and remains an open question.

**Contribution of the human perspective to the machine perspective.** Research findings from the human perspective can potentially have a strong influence on the machine perspective. An example from more general network visualisation is found in the work of Purchase et al. [Pur97, PCJ96] who confirmed the negative effects of edge crossings and bends on the readability of network layouts, which subsequently led to a rich body of literature in graph drawing on crossing and bend minimisation algorithms [Tam13].

The feedback link from empirical work on transit map usability to metro map algorithms is currently under‐developed. Nonetheless, findings from empirical studies that identify desired and un‐desired properties in transit maps, that correlate strongly with map usability, can provide key ingredients for new objectives in algorithmic map optimization. Similarly, if the effects of formal objectives (e.g., line straightness, edge lengths, edge orientations) that are part of many existing algorithms are confirmed in empirical studies, this would serve as a validation of these objectives in machine approaches.

### 6.3. Between Human and Design Perspectives

Interest in usability testing of schematised maps developed several decades after these were first created [Rob19a]. Over time, designers have developed and entrenched their own methodologies and principles for map creation, alongside beliefs about good practice. In tandem with this, individual network operators have also evolved their own requirements, e.g., for colour‐coding methods for individual lines. Hence there will inevitably be a natural tension between human versus design perspectives for the foreseeable future.

The belief amongst designers that octolinearity comprises a schematisation gold standard is evident from compilations by Ovenden [Ove03, Ove15]. However, beyond this, the diversity of solutions for all other aspects of design, including typography, topographical distortion, colour coding, and symbols for stations and interchanges, indicates the lack of anything approaching a consensus for just about every other aspect of creating schematised maps. At least one individual [Cer16] has advocated an international standardisation of basic parameters but there is little evidence of calls for unification elsewhere.

**Contribution of the design perspective to the human perspective.** The diversity of schematic mapping techniques and solutions certainly provides plenty of opportunities for researchers in the human perspective to investigate and resolve issues, but the potential contribution is far more deep‐seated because design creativity inspires hypotheses and theorising. For example, the five‐component framework for effective design [Rob14b, RGL17] was, in part, assembled as a result of a systematic design exploration of different levels of linearity [Rob12]. Similarly, the presence of different colour‐coding methods led to an analysis of perceptual versus cognitive aspects of route planning [LRR18]. Novel design techniques can focus attention in new ways and provide new methods to investigate psychological theories. Hence, concentric circles maps provided a tool by which the simplicity of line trajectories versus the overall coherence of a design could be compared [RNC16]. In all these cases, the key is that the design of maps resulted in more than merely evaluating the effectiveness of different versions, it also prompted a deeper attempt to understand the psychological concepts that underpin usability.

**Contribution of the human perspective to the design perspective.** The diversity of schematic mapping techniques and solutions, indicating fundamental disagreements between designers as to best practice, by itself would seem to demand evidence‐based approaches such as the usability testing research offered by the human perspective, and many relevant studies were discussed earlier. The most obviously important ones are those that directly investigated key design issues, such as the utility of different line colour‐coding [LRR18], and studies that investigate global layout, for example, comparing traditional octolinearity with more recently developed methods. Although attracting the attention of the media, at least one of these have been shown to have weaknesses [RNC16]. However, even with relatively clear findings and advice to offer, implementing such research is only part of the task: the dissemination of findings into multidisciplinary domains can be challenging.

An important category of studies comprises those that are user behaviour‐driven (as opposed to design hypothesis‐driven). For these, the decisions of the user are observed, and from this researchers work backwards in order to identify which aspects of the environment influenced them. In studies of route choice [Guo11, RMdG11] the network map proves to be an important component of the environment, with layout aspects of distance and directness having a role to play. In this specific case, however, the focus on the end product of the process (the decisions actually taken by the user) as opposed to the raw materials (e.g., how should a map be designed and colour‐coded) results in a different way of conceptualising the problem and a consequential focus on aspects of design that may have been overlooked by designers. Hence, on the list of criteria for effective design compiled by Ovenden, which were solicited from experienced designers [Ove08], not a single one refers to, or even implies, methods to influence route choice.

Undoubtedly, usability testing of maps will continue into the future, with the potential for further insights for designers as to how best to optimise the effectiveness of their work. However, it is also important for practitioners from both the design and human perspectives to be aware of the limitations as to what can be achieved by usability testing. As noted in Section 5.3.2, testing is most powerful when comparing and evaluating different prototypes but, for various reasons, it can be hard to identify generalisable design principles from these studies because of the difficulties in creating stimuli that are perfectly matched for all dimensions except the ones under investigation. To this should be added the problem that many design principles are somewhat subtle, perhaps contributing more to map aesthetics than usability, and hence are unlikely to generate sufficient effect sizes to be detectable statistically, even if they do genuinely have a slight part to play in design effectiveness. An example is the question of whether it is better to achieve a 90° bend using one single arc, or two linked arcs of 45° each.

Once design criteria move towards influencing aesthetics more than usability, they become subject to considerable individual differences, again limiting the extent to which they can be packaged as general design principles. However, individual differences in preferences for information presentation can have implications for usability, with formatting that is incompatible with preferences being detrimental to performance [RN01]. Providing multiple design formats to cater for individual differences in cognition seems to be a step too far for most transport organisations.

The consequences of the various issues mean that research needs to be carefully designed and targeted for maximum impact, with the focus on primary design issues that are most likely to influence usability. In cases where the impact of design decisions is more likely to be on aesthetics, it may be better to admit defeat and trust the experience and intuition of the designer rather than embarking upon a substantial research program. Overall, the human and design perspectives can be mutually supporting, but within limits. Also, there is a need to be vigilant for systematic individual differences in optimal information formats between different people. The best way to accommodate these is to offer the user a variety of journey planning methods, rather than prescribing one‐size‐fits‐all solutions.

### 6.4. Fusions of Design, Machine, and Human Perspectives

During our investigations in this survey, we established a vision on individual perspectives, together with their mutual relationships. Technology that meets users’ expectations still requires investigation in multiple disciplines, especially in the visualisation, algorithms, and psychology communities. According to our analysis, some papers classified in the machine perspective [NW11, WTLY12, WTH*13] cover all three perspectives to demonstrate the feasibility of proposed approaches. The techniques primarily followed a single‐directional process (Input → (**D**)esign → (**M**)achine → Output) in our model (Figure 3), then validating the approach by performing a user study (Output → (**H**)uman). The iterative process to improve the algorithm is often not documented, unfortunately. A task‐driven approach [MG19] follows the process (Input → (**U**)ser → (**D**)esign → Output), although an automatic approach along this line has not yet been developed. Pairwise perspectives have shown their strong connection in the previous sections, but any pair of perspectives alone is unlikely to achieve a bigger goal. Figure 3 in conjunction with our review invites us to revisit the definition of an idealised map and the corresponding methodology. We strongly believe that a more interwoven collaboration between the three perspectives not only will lead to superior results, but will be essential to substantially push forward the understanding and development of transit maps.

## 7. Research Challenges and Discussion

**Figure 21:**
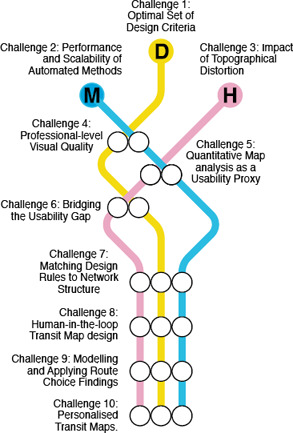
A transit map metaphor that shows the relationships between selected challenges, where each colour corresponds to one perspective (see Figure 3(b)).


The challenges listed below represent strategic topics and recurring issues. The transit map metaphor in Figure 21 provides an overview, with each perspective represented by a coloured line. The challenges are categorised by their dominant perspective(s) although each one is potentially relevant to all three.

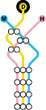
 **Challenge 1: Optimal Set of Design Criteria.** One major issue with the design perspective is that the principles are intuition‐derived rather than evidence‐derived. Sometimes, the hunches of designers drive what they do, which is why different designers do things so differently. A map in which all aforementioned criteria in Section 3 are targeted is not equal to a perfect design, since some criteria are mutually in conflict, or induce an over‐constrained and unsolvable problem. Finding a good combination of existing criteria or exploring a complete design space could give more insight into the relationships of various criteria. Some high‐level ideas, such as *a balanced map*, can probably be achieved by a combination of predefined low level criteria (e.g., minimising variability of edge length), but how?


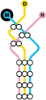
 **Challenge 2: Performance and Scalability of Automated Methods.** High‐performance techniques facilitate editing maps by incorporating user knowledge back to the process instantaneously, and scalable approaches allow us to manage large networks. Transit map problems are computationally hard, which implies that we cannot easily find efficient algorithms in general. One important aspect here is to recast this intractable problem and reformulate it as a more tractable one with additional constraints. Until now, it is hard to mutually compare the performance and quality of existing algorithms because there is no standardised format for recording transit maps, which makes it almost impossible to compare layout quality automatically. Crowd‐sourcing data management, such as the openMetroMap project [Ope], might help to fill this gap and also to record the provenance of datasets for systematic validation.


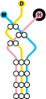
 **Challenge 3: Impact of Topographical Distortion.** The conflict between the objectives of simplified line trajectories versus minimising topographical distortion is a source of considerable disagreement in the creation and informal assessment of designs. Very little research has directly addressed this issue [BRB97], other than obliquely, in terms of the effects of map layout on route choice [Guo11] such that excessive topographical distortion can, in certain circumstances, detrimentally distort the implied relative efficiency of competing route options. The permissibility of topographical distortion is fundamental to determining criteria for effective schematisation, and given that these criteria should ideally be evidence‐based, research into understanding the impact of topographical distortion on the user must be a priority. This should concern not just route choices, but also the potential for, and consequences of, (1) disorientation if a layout deviates significantly from a user's mental model of a city, and (2) unintended inferences, which might be implied by relative positions of nearby or distant stations, and also of stations related to major landmarks that are included on the design. It is important to ascertain whether any disadvantages of topographical distortion apply generally, or only to particular regions of a map, for example, comparing densely versus sparsely served regions.


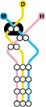
 **Challenge 4: Professional‐level Visual Quality.** The visual quality of machine‐generated transit maps is still not fully satisfactory [Rob19b]. One primary reason is the composition step sketched in Figure 2(a), which allows designers to arrange essential elements to produce visually pleasing maps. A precise formulation of every step in the design process is desirable, and has not yet been fully achieved. For example, *typography* is the sub‐field of making written text legible and readable. Font size is strongly correlated with the amount of information to be shown, which is often adjusted by designers, but is not intuitive for a computer. When designing a transit map, designers often decompose a long station name into multiple lines to simplify the labelling problem. This is a hard problem due to its combinatorial properties.


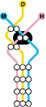
 **Challenge 5: Quantitative Map analysis as a Usability Proxy** Conducting customer and user surveys is a time‐consuming and expensive task that requires a lot of experience in the human perspective. However, researchers in the machine perspective are more often interested in the underlying algorithmic problems and are most familiar with evaluating their results using quantitative measures. Hence, identifying a set of quantitative measures for the usability of a transit map could be a promising interface. This requires that researchers from both perspectives identify quantitative measures which would then be validated as usability proxies by well‐defined user studies.


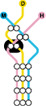
 **Challenge 6: Bridging the Usability Gap.** User‐acceptability of maps is an essential criterion for effective design, otherwise people will simply reject these and consult computerised journey planners instead. Therefore, it is important that the usability gap (Section 5.3.3) is not only understood but bridged. In other words, for researchers in usability testing, it would be desirable to identify measurable aspects of design effectiveness that correlate to at least some degree with user evaluations of this, so that maps are effective *and* preferred by users. In other words, measurements should also have *psychological utility* [Rob19c]. Route planning errors (choosing impossible routes) has psychological utility, but usability is potentially measurable in many different ways, and researchers need to explore more creative methods of objectively measuring map effectiveness. Hence, it has been suggested that route discriminability might be a useful measure of the ease with which users can identify a preferred route from amongst competing alternatives [Rob19c],


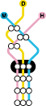
 **Challenge 7: Matching Design Rules to Network Structure.** With challenges to the *octolinearity* = *schematisation gold standard* conjecture [RNL*13] this lays open the possibility that various networks, structured in different ways, may require the use of different design rules in order for criteria for effective design to be optimised. Hence, certain networks may be naturally octolinear, hexalinear, tetralinear, curvilinear, or even based upon concentric circles and spokes [Rob12, NR18]. Forcing a network to comply with inappropriate design rules may result in complex line trajectories or topographical distortion, and also conflict with users’ mental models of a city. To a certain extent, designers can address this issue at the early stages of the creation process by viewing topographical maps and identifying the dominant shapes of the network, but this may only result in good solutions for relatively simple ones. Analysing the topographical input and detecting the most suited linearity system is an open challenge for the machine perspective. Further, the task of evaluating multiple maps created using a range of different design rules is one that is more appropriately tackled by researchers into automated layout [NN19].

The network structure can be further simplified into symbolic geometric objects in a hierarchical fashion by taking advantage of interactive visualisation. For example, a circular route can be abstracted and be represented as a circle to eliminate unnecessary details as a user zooms out of the map. In visualisation, this concept is called *visual abstraction*, which indicates a simplified form that visually summarises the subordinate structures as a whole. This facilitates users in forming a comprehensive understanding or overview of the area on the map [Cer16]. Nonetheless, the theoretical formalisation of the abstraction concept is in its infancy, not to mention its algorithmic methodology. This strategy requires map users’ experience or designers’ knowledge for better usability.


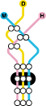
 **Challenge 8: Human‐in‐the‐loop Transit Map Design.** As mentioned in Section 6.1, interactivity is crucial in order to involve designers as partners in the automated creation process. The idea here is to ask machines to do work that they can do adequately, and preserve tasks that can be smartly done manually to designers. This requires investigation of what tasks are suitable for machines versus humans. Technically, how machines and designers can collaborate will be the main focus in this challenge. This would also include a broader set of information designers who wish to use the transit map metaphors to visualise other domains, such as abstract relationships in physics, biology, or engineering [WNTN19]. Transit maps, therefore, serve as visual metaphors representing an abstract information space, which leads to the challenge that a designer needs to find an appropriate mapping from data space to transit map space.


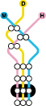
 **Challenge 9: Modelling and Applying Route Choice Findings.** There is now a substantial literature on the influence of configuration on route choice (Section 5.2.1), and a number of further relevant studies in which route choice has been investigated but not in the specific context of transit maps. The stage has surely now been reached at which it is possible, statistically, to model human route choice with respect to map layout, and validate models against actual route choice datasets where available. For individual networks, it would then technically be feasible to automatically audit and modify available maps with respect to (a) operational efficiency – for example, reducing the extent to which users distribute themselves between different options, which may contribute towards relieving versus causing overcrowding of individual routes [GZW*17, Xu17]; and (b) user efficiency – for example, altering the extent to which users are inadvertently prompted towards choosing inefficient routes [Guo11] or avoiding routes that are efficient but are implied to be inappropriate from map configuration [RMdG11]. Such steps are necessary to maintain the utility of transit maps in the face of alternatives such as digital journey planners.


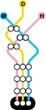
 **Challenge 10: Personalised Transit Maps.** The emergence of location‐based services and mobile devices has driven rapid development from static to dynamic and personalised maps. For topographical maps, numerous services have been established to support the user in routing tasks and finding nearby locations on small‐screen devices, but the development of such services for transit maps lags behind. In order to change this, all three perspectives are implicated, since it is far from clear how these services should be presented, how they should be technically realised and which of them are usable in practice. This problem becomes even more challenging in a dynamic setting. Changes in service patterns may occur, due to newly constructed lines, added or (temporarily) closed stations, or due to unexpected incidents interrupting some services temporarily. While the former can be implemented gradually, the latter need real‐time alterations. Changes in the transit map should clearly reflect the changes in service patterns, yet retain the users’ mental model of the corresponding map region.

## 8. Conclusions

The first schematic maps of transit networks appeared in the early 1930s, and the concept has spread to virtually all cities in the world. Strategies for transit map generation have been extensively studied from design viewpoints [Gar94, Ove03, Rob12] to practical usage [Rob19b]. The development of computational tools has the potential to facilitate designers to draft and also more objectively to evaluate their work. Research has also found direct practical applications, for example, the urban railway system in Karlsruhe, Germany [kvv] recently changed to curvilinear maps on the basis of generalisations from usability testing [RNL*13]. Another example is the Docklands Light Railway train map, which was adopted after testing of prototypes and commissioned by Transport for London [RR16]. Beyond static maps, mobile applications, with user intervention, have the potential to improve the navigation of complex networks through intuitive and straightforward representations. In consequence, a new scientific community has emerged and become active [smw], demonstrating an increasing interest in developing new techniques for schematic mapping.

## Acknowledgements

The project has received funding from the EU Horizon 2020 MSCA Grant No. 747985, from JSPS KAKENHI Grant No. 19H04120, and from the Austrian Science Fund (FWF) Grant No. P 31119.
